# Towards an integrated automatic design process for robot swarms

**DOI:** 10.12688/openreseurope.14025.2

**Published:** 2022-11-04

**Authors:** Darko Bozhinoski, Mauro Birattari

**Affiliations:** 1 IRIDIA, Université Libre de Bruxelles, Brussels, Belgium

**Keywords:** swarm robotics, integrated automatic design process, optimization-based design method, model-driven engineering, domain-specific languages (DSL)

## Abstract

**Background:** The specification of missions to be accomplished by a robot swarm has been rarely discussed in the literature: designers do not follow any standardized processes or use any tool to precisely define a mission that must be accomplished.

**Methods:** In this paper, we introduce a fully integrated design process that starts with the specification of a mission to be accomplished and terminates with the deployment of the robots in the target environment. We introduce Swarm Mission Language (SML), a textual language that allows swarm designers to specify missions. Using model-driven engineering techniques, we define a process that automatically transforms a mission specified in SML into a configuration setup for an optimization-based design method.  Upon completion, the output of the optimization-based design method is an instance of control software that is eventually deployed on real robots.

**Results:** We demonstrate the fully integrated process we propose on three different missions.

**Conclusions:** We aim to show that in order to create reliable, maintainable and verifiable robot swarms,  swarm designers may benefit from following standardised automatic design processes that will facilitate the design of control software in all stages of the development.

## 1 Introduction

In this paper, we make two original contributions: 1) we define a textual language for the specification of missions to be performed by a robot swarm and 2) we realize an engine that transforms a mission specification given in the aforementioned language into an objective function (and other configuration files) needed to automatically perform the design by optimization of a robot swarm that will accomplish the mission. These two original contributions, combined with an existing method for the design by optimization of control software for robot swarms ^1^, enable a fully integrated process that starts with the specification of a mission to be accomplished and terminates with the deployment of the robots in the target environment.

In swarm robotics, a large number of robots perform a mission that can not be accomplished by a single robot ^2, 3^. The collective behavior of the robot swarm is obtained through individual robots collaboration and cooperation. Hence, the collective behavior of a robot swarm is a result of the local interactions between the individual robot and its neighbors and its environment ^4^. In the general case, the complex nature of these interactions are virtually impossible to trace to the behaviour of the individual robots, which creates a gap between the collective behaviour that one wishes to obtain and what each of the individual robots should do. Bridging this gap is one of the main challenges in swarm robotics and the lack of a general methodology to bridging this gap influences how control software for robot swarms is designed and realized.

So far, robot swarms have been mostly designed manually: an individual-level behavior is iteratively improved and tested until a desired collective behavior is obtained. This non-systematic approach is neither reliable, nor consistent. The quality of the resulting solution strongly depends on the experience and intuition of the designer. To avoid, or at least reduce, the uncertainty induced by the crucial role of the human designer, automatic and semi-automatic design approaches have been proposed ^5– 8^.

Although a number of automatic approaches have been proposed in the software and system engineering literature ^9^, they have not been investigated in the context of swarm robotics. This is because these approaches, which focus on decoupling and automatizing the different phases of the robot life-cycle, appear to be inappropriate in swarm robotics. Indeed, they model the system to be realized at a level of abstraction that is too high and neglects the complex robot-robot and robot-environment interactions that characterize the operation of a robot swarm. For example, these approaches assume that it is possible to establish a mapping between high-level collective goals of the swarm and low-level individual behaviors of the robots comprised therein ^10– 12^. Unfortunately, the swarm robotics praxis indicates that making such a mapping explicit is not generally possible ^2, 3^.

For this reason, the most promising approaches that have been proposed so far for the automatic design of robot swarms are in the area of design by optimization ^13^. In design by optimization, the design problem is re-formulated into an optimization problem: an optimization algorithm searches a space of candidate solutions to maximize an objective function. In the context of the application of design by optimization to swarm robotics, a candidate solution is an instance of control software and the objective function is a mission-dependent metric that measures the performance of the swarm on the given mission ^3^. Depending on whether the design phase happens before or after the deployment of the software on the robots, we can distinguish between two classes of design methods ^14^: off-line and on-line. In this work, we focus on off-line automatic design ^15^, although the proposed ideas could be adapted to on-line design as well. Within an off-line design process, the performance of candidate designs are assessed by an optimization algorithm typically via computer-based simulations. After the optimization algorithm terminates, the selected design is deployed to the individual robots and the swarm is placed in its target environment.

In this paper, we present a first instance of a fully automatic and integrated process for the design of collective behaviors for robot swarms. The novel contribution that enables this integrated process is the definition of a formal and systematic approach to the specification of missions to be accomplished by a robot swarm. This approach is rooted in model-driven engineering ^16^: a research direction in software engineering that aims at simplifying the design, implementation, and realization of complex software systems by shifting the designer’s attention from code to models. Recently, model-driven engineering has been often used in the design of robot systems as it dispenses the designer from reasoning on complex robot behaviours at the code level, which is cumbersome and undesirable ^17^. Indeed, in model-driven engineering, models are expressed at an appropriately high level of abstraction using domain-specific languages with concepts that are close to the problem domain and not directly bound to the robotic platform at hand. This makes the realization of complex systems manageable as models are easier than code to specify, understand, and maintain ^18^. Specifically, in this paper we present and demonstrate an integrated automatic design process for robot swarms that, starting from requirements specified in a textual language, generates code and deploy it on real robots. We introduce a language that we call swarm mission language (SML) which allows one to specify missions to be accomplished by a robot swarm. In the paper, we present a first implementation of SML that supports the specification of missions in which rewards and penalties can be expressed with reference to regions: that is, rewards and penalties are computed according to whether robots (and/or relevant objects) are in a particular part of the environment at specific moments in time. Many of the most studied swarm robotics missions like aggregation, foraging and collective exploration can be modeled through the concept of a region. Furthermore, we develop an engine that translates a range of missions specified in SML into all the resources needed to launch Chocolate ^1^, a state-of-the-art automatic method for the off-line design of robot swarms ^19^ that, using simulation performed by ARGoS3 ^20^, produces control software that can be directly ported to e-puck robots ^21^. To demonstrate SML and the integrated automatic design process, we specify three missions and we automatically generate the control software that allows a swarm of e-puck robots to accomplish them.

## 2 Related work

### 2.1 Automatic off-line design of robot swarms

In swarm robotics, neuro-evolutionary robotics ^22, 23^ is the most studied automatic design approach. In neuro-evolutionary swarm robotics ^6^, each individual robot control software is a neural network. The parameters of the neural network are obtained via an evolutionary algorithm that optimizes a mission-specific objective function taking sensor readings as an input and returning actuation commands as an output. A large literature shows that neuro-evolutionary robotics is able to produce robot swarms that can perform well in a variety of missions ^4, 24^. However, the neuro-evolutionary approach does not appear to be able to scale in complexity for realistic robot swarm applications. One of the main causes is the difficulty to overcome the so called reality gap ^4^. The reality gap is the discrepancy between reality and the simulation models used in the design process ^25^. Because of the reality gap, the performance of control software developed in simulation typically drops when the control software is ported to the real robots. It has been argued that this drop in performance is the result of a sort of overfitting of the obtained solution to the particular conditions encountered during the design process ^25– 27^.

AutoMoDe ^7, 28^ is an alternative approach that deviates from traditional neuro-evolutionary robotics. It aims to address one of the main concerns in neuro-evolutionary robotics and that is the reality gap due to the excessive representation power of neural networks. Inspired by the notion of bias-variance tradeoff ^29^, AutoMoDe produces control software with restricted representational power. It does so by selecting, combining, and fine-tuning a set of predefined modules. AutoMoDe is a general, abstract framework. To define a design method that can be used to design control software, AutoMoDe must be specialized to the specific platform at hand, as formally described by a reference model. Also, a number of elements need to be defined, including the optimization algorithm to be adopted, the modules that will be used by the optimization algorithm, and the architecture into which the predefined modules should be combined. Up to now, AutoMoDe has been specialized for a specific version of the e-puck robot; the optimization algorithms that have been adopted are F-Race ^30, 31^, Iterated F-Race ^32^, and simulation annealing ^33^; and the architecture in which modules have been combined are probabilistic finite-state machines ^1, 34^ and behaviour trees ^35^. Chocolate is a state of the art automatic design method from the AutoMoDe family that achieves significantly better results in crossing the reality gap than neuro-evolutionary approaches ^1^. It uses Iterated F-Race as an optimization algorithm and probabilistic finite-state machines as a control architecture.

An aspect that is rarely discussed in the literature is the specification of the mission for which the automatic design method must generate control software ^3^. Designers of robot swarms do not follow any standardized processes or use any tool that precisely defines the mission to be accomplished. For example, the aforementioned ARGoS simulator enables specifying missions through a combination of XML files and loop functions defined in C++ ^20^. It provides a great deal of flexibility for designers to design missions the way they prefer. Designers can incorporate a rich variety of elements related to the operational context or the characteristics of the robots. However, it is a tedious process to manually specify all elements of a mission to be performed by a robot swarm without following any predefined process. This might create situations where designers use environmental elements that are important to obtain a desired collective behaviour for one application scenario, while omitting the same elements if they impede the desired collective behaviour. This *ad-hoc* mission specification process might create confusion between designers that are working on a same set of missions. Moreover, if requirements are not defined explicitly, it is impossible to check the consistency of mission models. It is also impossible to tell whether a robot swarm eventually performs the mission successfully or not. To simplify the communication between designers and to check for possible inconsistencies, all these aspects must be formally defined and automatized.

### 2.2 System and software engineering for robotics

System and software engineers have made their contribution to robotics by providing tools and standardized methodologies for the specification and the definition of robot systems ^36^. In system and software engineering, researchers have addressed a variety of emerging challenges in the design and development of complex systems by providing generic solutions, often disregarding their specific nature. One of the main challenges is collecting requirements ^37^. To simplify the requirements’ elicitation for complex system, researchers have focused on defining standard processes and methods that can be fully or partially automatized.

In requirements engineering, there is a basic assumption that underlines most of the approaches: if all requirements are known, it is always possible to decompose any high-level goal into a sequence of operations that allow the system to attain it. For example, goal orientation ^38^ is a widely recognized process for eliciting, modeling, specifying and analyzing system requirements. Goals are statements of intent organized in AND/OR structures that can range from high-level strategic concerns to low-level technical requirements and assumptions on the system and the environment where it operates. It is generally accepted that robotic systems are too complex for engineers to obtain complete requirements on the system and the environment ^37^. Hence, it is typically assumed that robotic systems are highly uncertain due to incomplete requirements. However, this is not a valid assumption to be made in swarm robotics. The uncertainty of a robot swarm is not only the result of incomplete requirements, but it mostly emerges from the complex interactions between the robots and between the robots and the environment. This means that the gap between the high-level swarm goals and the low-level robot behaviours is inevitable.

In a number of works ^10– 12^, system and software engineers have synthesized low-level robot behaviours from high-level mission descriptions. For example, FLYAQ ^11^ is a tool that allows defining missions for teams of multi-copters. Starting from a high-level description of the mission, FLYAQ automatically generates a detailed flight plan for a team of autonomous multicopters that can perform the specified mission, while preventing collisions between multicopters and obstacles. FLYAQ was developed based on model-driven engineering principles. It uses a family of domain-specific languages for specifying civilian missions for multi-robot systems ^10^. Each language focuses on a certain aspect of the system:

- *Monitoring modeling language:* a language that enables the specification of the mission goals complemented by the definition of the context in which the mission will be realized.
- *Robot language (RL):* a language to specify the type and the configuration of the robots that will be in charge of realizing the specified mission.
- *Behaviour language (BL):* a language that specifies robot atomic movements and actions.

However, FLYAQ cannot be used in swarm robotics because the nature of robot swarms does not support the synthesis of the individual robot behaviour from the collective swarm behaviour.

To the best of our knowledge, requirements specification for swarm robotics has not been properly addressed as a research question. The highest level of abstraction that has been extensively discussed in swarm robotics is the development process. A work in this direction is Buzz, a scripting language for programming heterogeneous robot swarms ^39, 40^. The language offers primitives to define swarm behaviors, both in a bottom-up and in a top-down fashion. The formal specification of requirements for robot swarms was partially discussed by Brambilla *et al*. ^41^ in a work devoted to a top-down design approach based on prescriptive modeling and model checking. The approach of Brambilla *et al*. consists of four phases to specify, design, realize, and validate a robot swarm. In the first phase, the developer specifies the requirements using temporal logic. However, the approach does not provide a precise process definition of requirements specification but rather a set of examples on how designers can use probabilistic computation tree logic to specify swarm-level requirements.

We believe that model-driven engineering can provide support in gathering explicit and clear requirements for robot swarms. Model-driven engineering has been explored in the design of complex systems being an essential factor in reducing costs and development time. It has been successfully used in various domains including avionics, automotive, and telecommunications ^16^. For example, domain-specific modelling (DSM) ^42^ is a powerful methodology in model-driven engineering, which enables users to model systems using concepts close to the problem definition.

## 3 Integrated automatic design process for robot swarms

**Figure 1.**
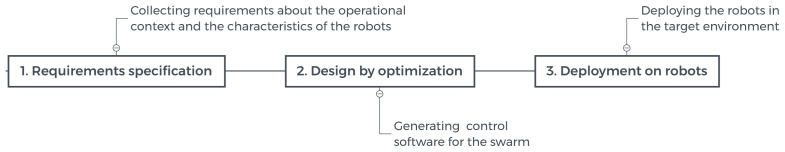
Automatic off-line design of robot swarms.

We present here the main phases and key activities to design control software for a robot swarm in a systematic way ( Figure 1). In an automatic off-line design process, we identify three phases: requirements specification, design by optimization, and deployment of the control software on the robots.

In *requirements specification*, the designer identifies and declares all the characteristics of the robot swarm, the target environment in which it will operate, the mission that it should accomplish, the objective that it should fulfil, the possible constraints, etc. In the current state of the art, no standard process has been defined for collecting requirements. Typically, designers specify missions informally and in an *ad-hoc* manner, which makes specifications vague and eventually hinders a final verification of whether the swarm developed satisfies the requirements or not ^43^. Starting from the requirements, the designer defines an objective function to be then optimized in the second phase. As requirements are specified informally, this step must be performed manually and is discretionary, non-repeatable, and error prone. In the second phase, *design by optimization*, the control software of the individual robots comprised in the swarm is produced by an automatic design method ^14, 15^. An automatic design method is defined through: (i) a reference model of the robotic platform for which it can design control software; (ii) an optimization algorithm; and (iii) the space of control software it can possibly produce. The reference model is an abstraction of the robotic platform that specifies in formal terms the characteristics and capabilities of the robots; the optimization algorithm is the algorithm that drives the optimization process; and the space of the control software that can be produced is typically expressed by a parametric architecture and by the set of the possible values of its parameters.

The last phase is the *deployment on the robots*. It consists of all the activities related to the transfer of the control software produced to the robots in the target environment. Some tools exist that are able to generate code that can be directly ported to the robots. For example, ARGoS ^20^, a multi-engine simulator for robot swarms, can currently generate code for a number of platforms including marXbot ^44^, e-puck ^21^, Thymio ^45^, Kilobot ^46^, and Khepera IV ^47^. Due to its modular nature, ARGoS can be extended to generate code for a variety of robotic platforms.

### 3.1 Requirements specification for robot swarms

**Figure 2.**
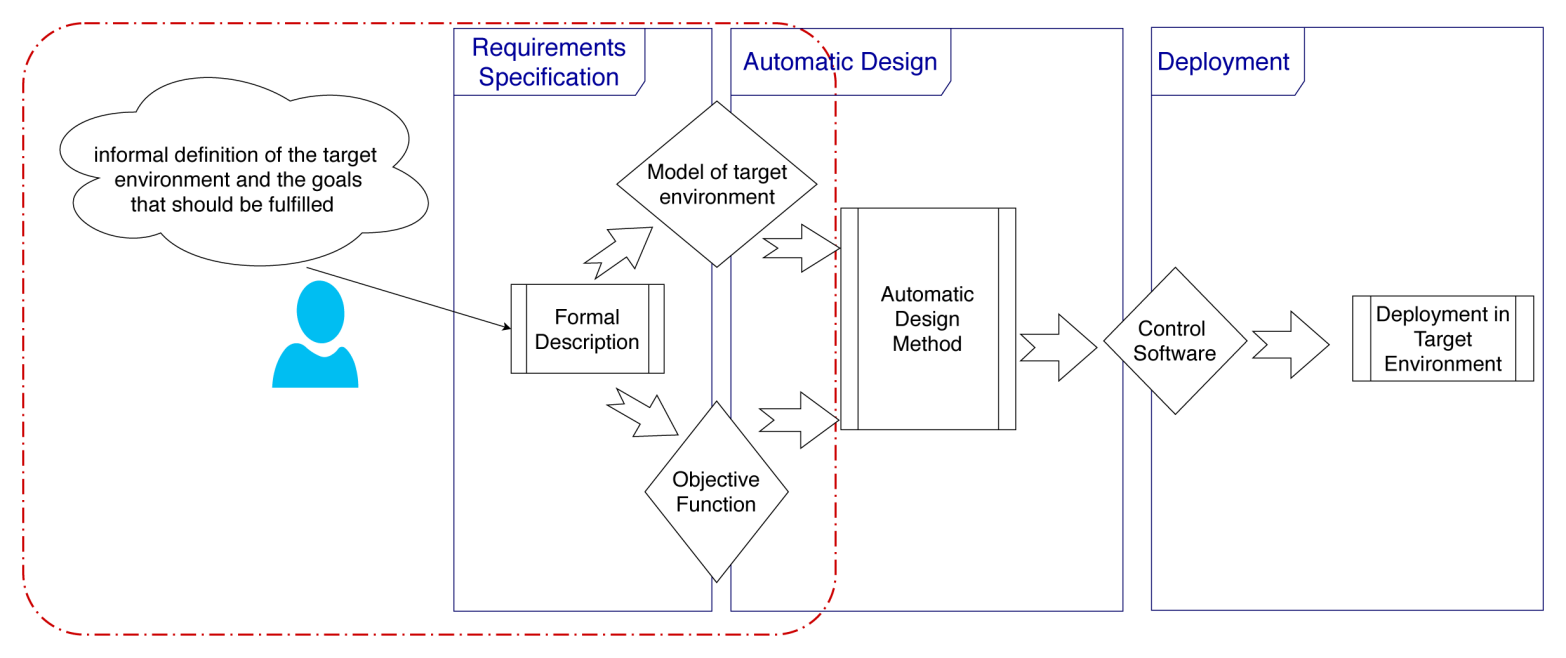
Integrated automatic design process for robot swarms.

We present an approach that enables experts (control software designers) and non-expert users (non-technical end users) to specify requirements for robot swarms in a standard and consistent way. Figure 2 depicts the workflow of the automatic design process. The novelty of our approach is the requirements specification phase, outlined by the dash-dotted line in Figure 2.

The user has an informal picture of what the robot swarm should do and of the environment in which it should operate. From these informal requirements, a formal model of the mission goals and the target environment should be defined. We developed swarm mission language (SML) to allow users to specify requirements. The output of this phase is an objective function that will be subsequently optimized by the optimization process, and a model of the target environment to be used in the simulations performed within the automatic design process. The model of the target environment is specific to the automatic design method and the tools used. In our work, we used model-to-model transformation techniques to translate the missions specified in SML into configuration files for Chocolate.

### 3.2 Design by optimization

In the subsequent automatic design phase, we use Chocolate with a design budget of 200K simulation runs as an automatic design method to generate control software for the robot swarm. Chocolate operates on a set of six low-level behaviors and six conditions ^1^. In this context, a low-level behavior defines how the robot operates its actuators in response to the readings of its sensors. On the other hand, a condition is an event that the robot perceives via its sensors and that determines whether the robot should transition from one behavior to another. Conditions contribute to determine which behavior is executed at any moment in time.

The low-level behaviors on which Chocolate operates are the following.

*Exploration:* the robot moves straight forward, if the front of the robot is clear of obstacles. If an obstacle is perceived via the front proximity sensors, the robot turns in-place for a random number of control cycles drawn in {0, ..., *τ*}, where *τ* is an integer parameter ∈ {0, ..., 100}.

*Stop:* the robot stops its movement.

*Phototaxis:* the robot moves towards a light source. The robot moves forward while avoiding obstacles, if it does not perceive any light source.

*Anti-phototaxis:* the robot moves away from a light source. The robot moves forward while avoiding obstacles, if no light source is perceived.

*Attraction:* the robot moves towards its neighboring peers, following *αV _d_*, where *α* ∈ [1, 5] controls the speed of convergence towards the peers. The robot moves straight forward while avoiding obstacles, if it does not perceive any peer.

*Repulsion:* the robot moves away from its neighboring peers, following – *αV _d_*, where *α* ∈ [1, 5] controls the speed of divergence. The robot moves straight forward while avoiding obstacles, if it does not perceive any peer in its neighborhood.

The conditions under which a robot switches from a behavior to another are the following.

*Black-floor:* true with probability *β*, if the ground situated below the robot is perceived as black.

*Gray-floor:* true with probability *β*, if the ground situated below the robot is perceived as gray.

*White-floor:* true with probability *β*, if the ground situated below the robot is perceived as white.

*Neighbor-count:* true with probability *z* ( *n*) = (1 + *e ^η^* ^( *ξ−n*)^) ^–1^, where *n* is number of detected peers. The parameters *η* ∈ [0, 20] and *ξ* ∈ {0, ..., 10} control the steepness and the inflection point of the function, respectively.

*Inverted-neighbor-count:* true with probability 1−z ( *n*).

*Fixed-probability:* true with probability *β*.

For more details on the low-level behaviours and conditions of Chocolate, we refer the reader to their original description ^7^.

## 4 An approach to specifying swarm missions

In this section, we present an approach to specifying swarm missions. To be able to specify missions, we need to understand the nature of the requirements in swarm robotics. First, we discuss a classification framework for swarm missions, then we develop a metamodel that defines the semantics of the Swarm Modeling Language (SML).

### 4.1 Classification framework for swarm missions

A classification framework of the main missions studied in the literature has already been proposed ^4^. Missions have been classified in different categories: spatially organizing missions, navigation missions, collective decision-making, and other swarm missions.

Spatially organizing missions focuses on organizing and distributing robots and objects in the environment. This category consists of missions like aggregation (robots group in a region of the environment), pattern formation (robots position themselves on a regular lattice), chain formation (robots position themselves so as to connect two points in the environment), self-assembly and morphogenesis (robots physically connect to each other following a particular pattern), and object clustering and assembling (robots position objects in the environment).

Navigation missions focus on coordinating the movements of a swarm of robots. The following missions are part of this category: collective exploration (robots explore an unknown environment), coordinated motion, also known as flocking (robots move in formation similarly to schools of fish or flocks of birds), collective transport (robots cooperate to transport an object).

Collective decision-making is a set of missions where the focus is on how robots influence each other when making choices. Here, we can find missions like consensus achievement (robots reach a consensus on one choice among different alternatives) and task allocation (robots dynamically choose the task to execute in order to maximize performance). The last category is for missions that are outside the scope of the previous classes. Here, we can find missions like collective fault detection (robots autonomously detect failures and faulty behaviors) and human-swarm interaction. This classification framework is interesting to understand the different types of collective behaviours robots can perform. However, one important aspect that has not been discussed in this framework is how do we tell whether the swarm accomplishes its mission and how we quantify the degree to which it is successful. In this work, we classify missions in terms of the objective function that describes them. That being said, we propose a different classification framework based on a measure of success for the mission. In many of the missions mentioned above, the typical way to measure success is through the concept of a region. Rewards and penalties can be naturally given according to whether robots (and/or relevant objects) are in a specific region at a certain moment in time, or not.

### 4.2 SML metamodel

**Figure 3.**
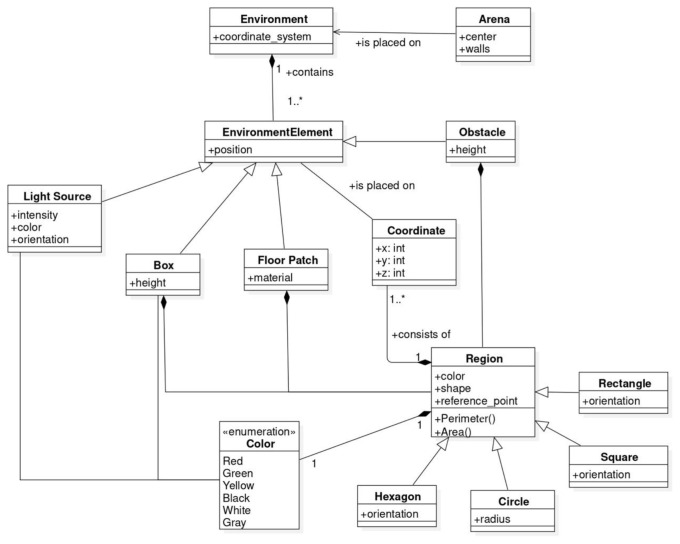
Constructs to specify the environment.

**Figure 4.**
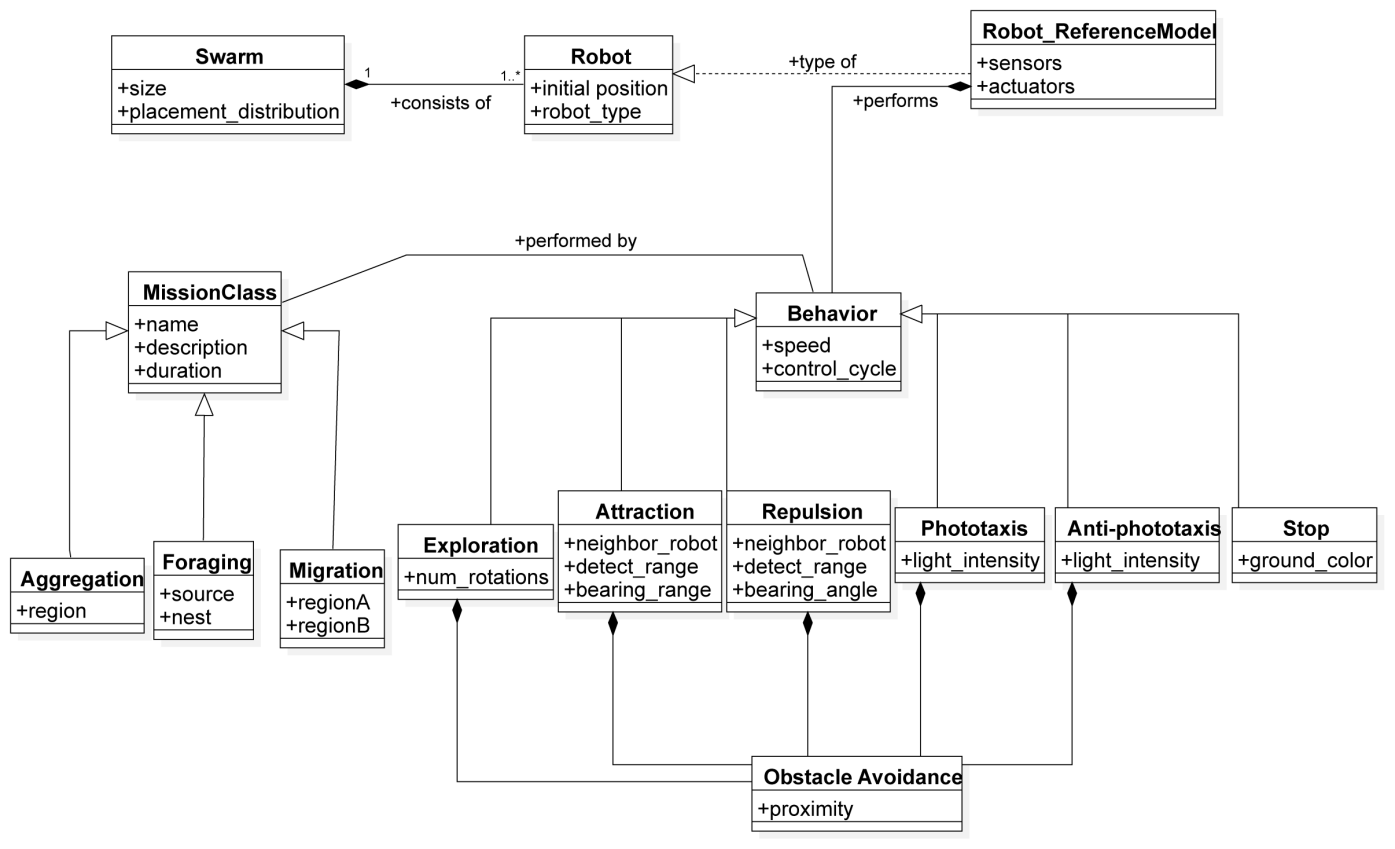
Constructs to specify the overall robot swarm, individual robot behaviour, and the mission class.

**Figure 5.**
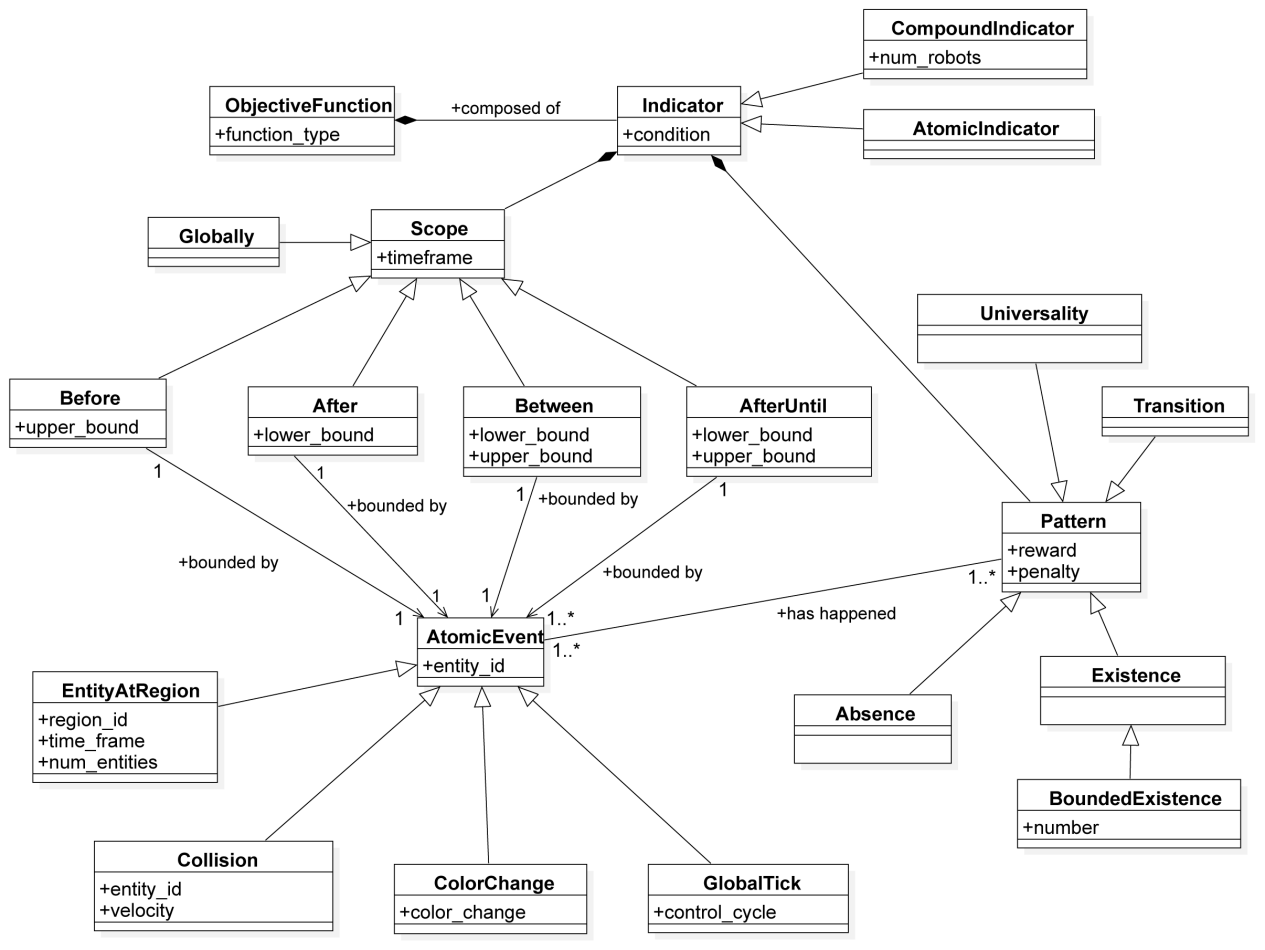
Constructs to specify the assessment of the mission objectives.

In this section, we describe the SML metamodel on which we base the Swarm Mission Language. In Figure 3, Figure 4, and Figure 5, we present the concepts used to design the language. The proposed abstractions are tailored to the literature in swarm robotics, as discussed in the previous section. With them, we intend to provide a way to define missions in a standard and consistent way. We formally define a mission as follows.

**Definition 4.1** (Mission). A mission is a triplet *M* = ( *E*, *S*, *O*) where:

- *E* is the environment where the mission is performed;
- *S* is the robot swarm that should perform it;
- *O* is the objective function to be optimized.

The SML metamodel provides modeling constructs that enable the specification of the three aspects: the environment, the robot swarm and the objective function.

The environment where the mission is performed is defined through the *Environment* construct. It consists of an *Arena* that gives the context of the mission. The *EnvironmentElement* is an abstract metaclass that can be implemented through the classes of the various elements placed in the environment. The number and type of elements that can be used across missions might vary. The central concept in SML is the one of *Region*. The *Region* is an abstract class that can be instantiated as a specific geometric shape referring to coordinates in the environment. All environment constructs can be found in Figure 3.

The *Swarm* is a construct that is developed to specify the number and type of robots performing the mission, their initial position, and the set of low-level behaviours they can perform. The *Robot* construct represents a specific instance of the reference model of a robot.

**Definition 4.2** (Reference Model). A reference model of a robot is a tuple *R* = ( *T*, *A*, *P*, *V*, *Z*) where:

- *T* is a set of actuators and sensors;
- *A* is a set of attributes;
- *P* : *T →A* is an assignment function that maps sensors/actuators *T* to the corresponding attributes *A*;
- *V* is a set of values that can be given to the attributes;
- *Z* : *A →V* is an assignment function for the attributes.

An example of a reference model is shown in Table 1.

**Table 1.** Reference model RM 1.1 ^48^. Sensors and actuators of the extended version of the e-puck robot.

sensor/actuator	variables	values
proximity	*prox _i_*, with *i* ∈ {0, ..., 7}	[0, 1]
light	*light _i_*, with *i* ∈ {0, ..., 7}	[0, 1]
ground	*ground _i_*, with *i* ∈ {0, ..., 2}	{ *black, gray, white*}
range-and-bearing	*n*	{0, ..., 19}
	*V _d_*	([0, 0.7]m,[0, 2 *π*] radian)
wheels	*ν _l_, ν _r_*	[–0.12, 0.12]ms ^–1^

The *Behaviour* is an abstract class that represents a low-level action that an individual robot can perform. A behaviour can be instantiated on a different level of abstraction and can be modular, as in our implementation. The modular structure of the Behavior construct of the language allows composition of atomic sub-behaviors into complex behaviours. For example, in Chocolate ^1^ we implemented an Obstacle Avoidance behavior as a sub-behaviour into five low-level behaviors. *Behavior* is associated with the concept of *MissionClass*, an abstract class that presents a set of missions. The association between the behavior and mission class relates to the corresponding mapping that defines which behaviors are suitable for a specific mission class to be performed. The *MissionClass* is a construct that provides a template for a set of missions that share similar behavior patterns. In this work, all implemented behaviors were used to create a swarm controller for each instantiated mission class. We instantiated three mission classes: aggregation, foraging and migration.

The third aspect of the language is the representation of the objective function that is used to measure the success of the mission execution ( Figure 5). The *ObjectiveFunction* is a construct that consists of a set of *Indicators*. An *Indicator* is an abstract class that represents the smallest measurable unit of performance in a mission. It can take one of the two forms:

- *Atomic Indicator*: a construct that represents the smallest measurable unit of performance for an individual robot;
- *Compound Indicator*: a construct that represents the smallest measurable unit of performance for a set of robots.

The idea of the indicator is taken from Dwyer *et al*. ^49^ and Autili *et al*. ^50^. Indicators are defined as a pattern in a scope—i.e., each indicator is represented through two constructs:

- *Pattern*: a construct used to measure the degree of success in a mission;
- *Scope*: a timeframe within the duration of the mission during which the pattern is quantified.

We identify the following scopes: *GLOBALLY*, *AFTER* the occurrence of an event, *BEFORE* the occurrence of an event, *BETWEEN* the occurrence of two events, or *AFTER* the occurrence of one event, and *UNTIL* the occurrence of an another one ( *AFTER UNTIL*). *Atomic Event* is an abstract class that specifies the possible events that can happen during a mission. In our work, we identified four abstract events:

- *Color Change*: an event that is triggered when there is a color change in the mission entities;
- *Entity at Region*: an event that is triggered when a set of entities are in a specific region (e.g., robots stay in a certain region);
- *Collision*: an event that is triggered when two entities in the mission collide. For example, it might be a collision between two robots, a collision between a robot and an obstacle, a collision between a robot and a wall in the arena etc.;
- *Global Tick*: an event that is triggered at every step of the mission execution.

As mentioned before, patterns are mission-agnostic concepts that are used to quantify the success of a mission. We identify a set of patterns that quantifies the appearance of a specific mission concept during execution. In the following, we describe three patterns of SML:

- *Absence*: a pattern that quantifies the absence of an event—e.g., a robot is not in a specific region, a robot does not perceive light, etc.;
- *Existence*: a pattern that quantifies the existence of an event—e.g., a robot is in a specific region, a robot broadcasts a message, etc.;
- *Universality*: a pattern that quantifies the universality of an event—something that should always occur.
- *Transition*: a pattern that quantifies the transition between two events—e.g.a robot moves from one region to another.

*Bounded existence* is a sub-pattern of an existence which quantifies the existence of an event only in certain bounds (something should occur at most *n* times).

These constructs are abstract and need to be instantiated to be realized in SML. More details about the instantiation of these constructs is given in Section 5.

## 5 Implementation of SML as a textual domain specific language (DSL)

The SML Language conforms to the SML metamodel discussed in Section 4.2. We implemented SML as a textual domain specific language (DSL) to enable non-technical end users (users that do not necessarily have technical knowledge in swarm robotics) and swarm designers to specify missions using structured English grammar. The development of SML is based on the following concepts:

- **Extensibility**:Control software designers should be able to add new constructs for new classes of missions. One of the most important goals in the development of SML was to provide an easy way for designers to add new language constructs. This enables reusability of the language across projects, missions, and research groups.
- **Variability**: Control software designers should be able to define missions in a variety of ways. Variability plays an important role in the definition of swarm missions. There are many examples in the swarm robotics literature where the position of the robots and of other objects in the environment is defined in a probabilistic way. There are many other examples where the position of the objects is provided in a deterministic way. Providing a rich interface using variability points was the second most important goal in the design of SML.
- **Usability**: Non-technical end users (users that do not necessarily have technical knowledge in swarm robotics) should be able to specify missions.
- **Generality**: Control software designers should be able to specify classes of missions that are non-trivial. Having constructs that are generic and independent from the functional behaviour of the robots is extremely relevant for managing the complexity of the missions to be performed.

### 5.1 SML syntax

**Figure 6.**
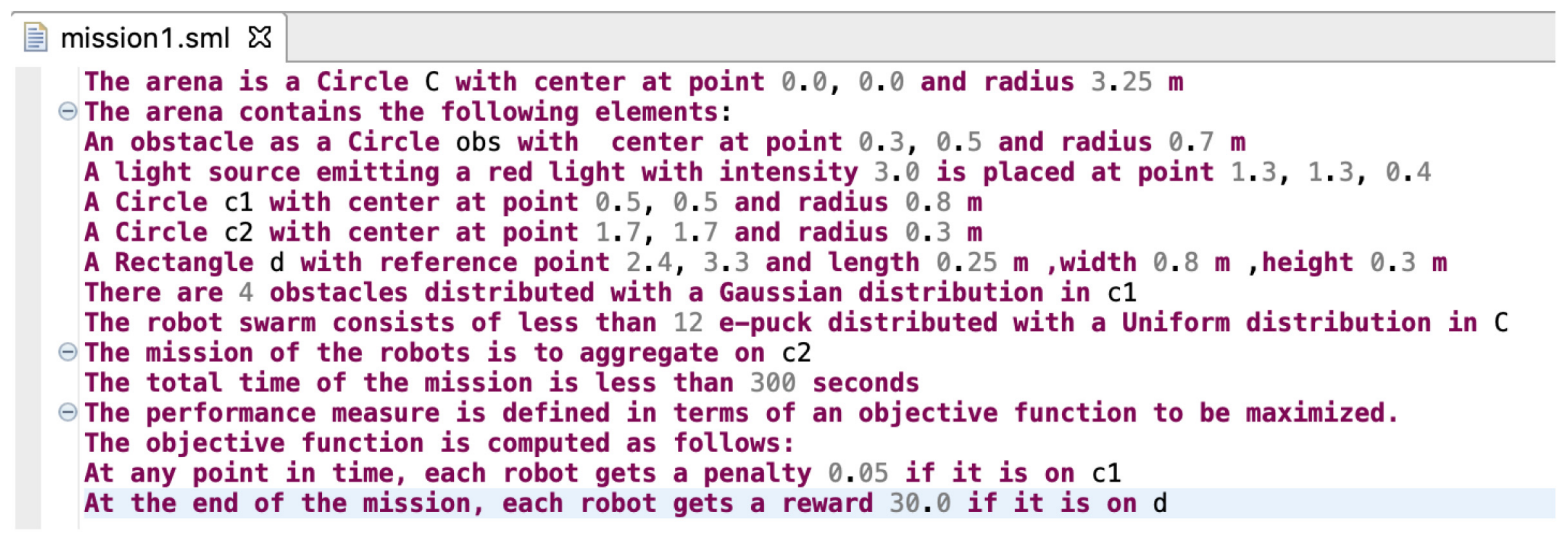
Screenshot of the Swarm Mission Language (SML) editor in Xtext.

To implement the language we used Xtext ^51^. Xtext is a framework for developing domain-specific languages. It provides a full infrastructure, including parser, linker, typechecker, and compiler. The current implementation of SML is focused on realizing abstract concepts through a set of elements that are necessary to define a mission. We will extend the set of these elements in a future work, which will increase the application domain of SML. It is important to note that the current implementation of SML in Xtext includes variable name resolution, parse error visualisation, and syntax highlighting. A screenshot of the SML editor is shown in Figure 6.

Snippet code of the grammar that enables mission description and swarm configuration is presented in Listing 1. In the implementation, the model of the language is realised through three high level concepts: *Environment* specification, *Swarm configuration*, and *Mission objective* specification. The current implementation of the language supports three different types of mission: *aggregation*, *foraging*, and *migration*. In aggregation, the robot swarm must group ( Figure 8). In foraging, the swarm must collect items from the environment and brings them to the nest ( Figure 9); while in migration, the swarm must move from one initial location to another one ( Figure 10). The current implementation of the language is extensible in the following directions: (i) it allows new classes of missions to be defined through the *Task* construct and (ii) it allows new types of robots to be defined through the *Robot* construct. The current implementation includes support for e-puck ^21^ and s-bot ^52^. Each of these robots has different sensors and actuators with different attributes and values.

**Listing 1.**
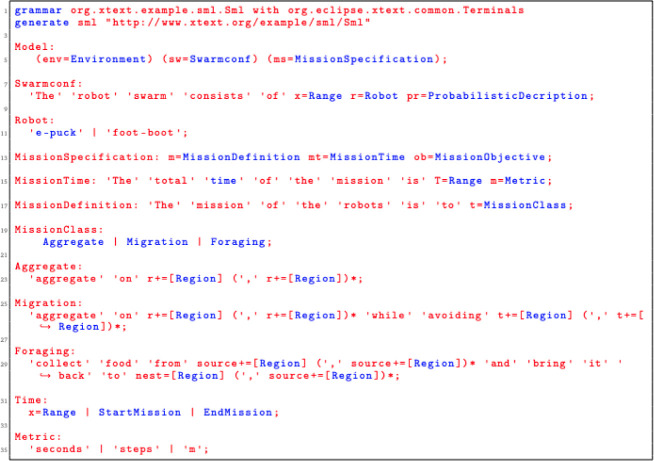
Language grammar for defining the mission and the swarm configuration.

Snippet code of the grammar that enables the specification of mission objectives is presented in Listing 2. In the current implementation of the language, *Occurence* is the only pattern that we used to quantify the success of the mission. Through this pattern, we can specify a variety of missions that are non-trivial. We satisfy one of the main aspects of our language mentioned in Section 5—generality. Patterns enable generality by separating the concepts on how the success of the mission is measured from the functional behavior of the robots. As we identify additional patterns in robot swarm missions, we plan to add them as additional constructs in SML. SML is highly extensible, which allows new pattern constructs to be added at a later stage of the development.

**Listing 2.**
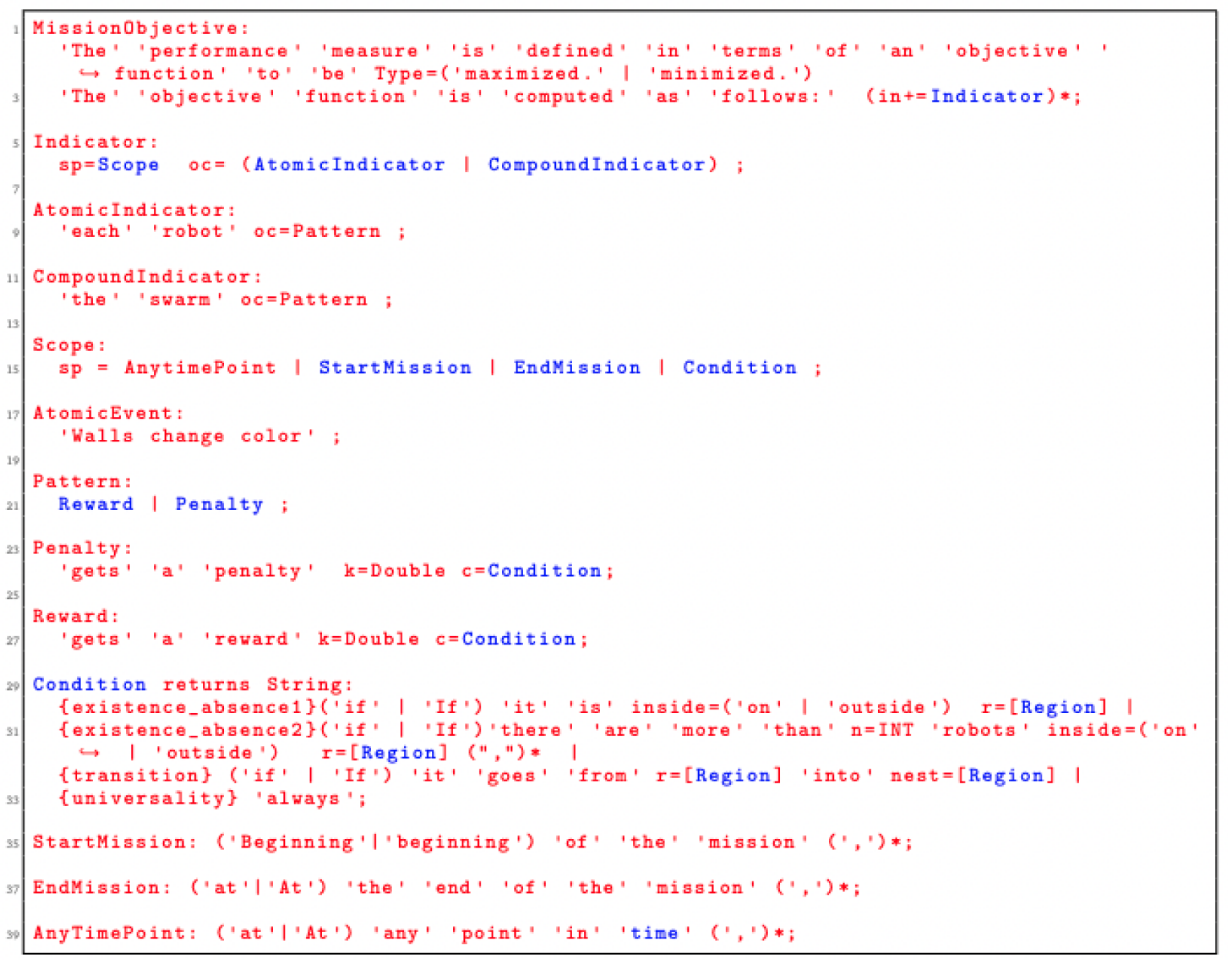
Language grammar for defining mission objectives.

Patterns need to be instantiated. *Occurence* is instantiated through the concepts of *Reward* and *Penalty*. *Condition* is another construct that plays an important role in the definition of the language. We use it to determine the situations under which a score is assigned to the swarm that executes the mission. This construct can be extended by identifying other situations that are relevant in swarm robotics. In the current implementation, a score is assigned only through the concept of a region: whether a robot, a set of robots, or objects are in a particular part of the environment.

Snippet code of the grammar that enables the specification of the environment elements is presented in Listing 3. We implemented a set of environmental elements that can be directly used in missions. At the moment of writing, we have implemented four environmental elements: *Wall*, *Light Source*, *Floor Patch* and *Obstacle*. Each of these elements has been defined through different attributes. The extensibility of SML allows designers to easily add new environmental constructs if they need them.

**Listing 3.**
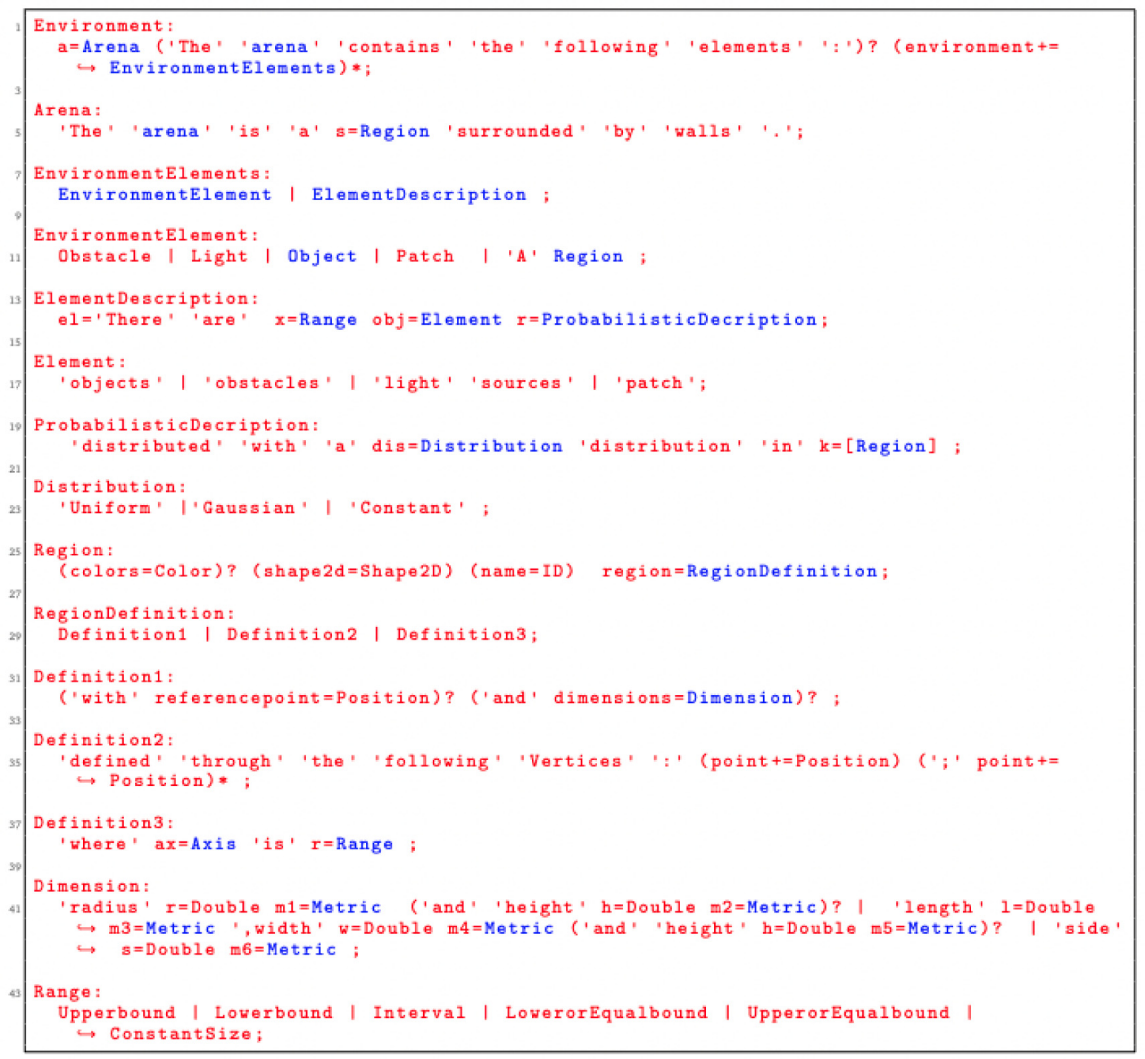
Language grammar for defining the environment.

To demonstrate the variability of SML, we defined a set of variability points for the different constructs in SML. These variability points allow designers to formulate mission concepts in a variety of ways. For example, the position of robots, objects, and obstacles in the environment can be specified either in a deterministic or a stochastic way. In Figure 6, we present a simple mission specification. An obstacle is defined in a deterministic way with the following statement:

*The arena contains the following elements:*

*An obstacle as a Circle obs with center at point 0.3, 0.5 and radius 0.7 m.*

An example of obstacle description in a stochastic way is presented through the following statement:

*The arena contains the following elements:*

*A Circle c1 with center at point 0.5, 0.5 and radius 0.8 m.*

*There are 4 obstacles distributed with a Gaussian distribution in c1.*

This aspect of the language provides a rich platform for the definition of the various elements in the environment. *Region* is the basic language construct we use to define the various elements. In the current implementation of SML, we have three variability points on how we specify regions:

- through one reference point and set of dimensions (Definition1: Figure 9);
- only through a set of reference points (Definition2: Figure 9);
- through the global coordinate system (Definition3: Figure 9).

We made the assumption that a global coordinate system exists and its origin is positioned in the center of the arena. This coordinate system is only used to specify the position of elements in the environment. It is important to note that robots are unable to utilize this coordinate system to position themselves or place objects in the arena because usage of a global coordinate system to create a certain robot behaviour goes against the main principles of swarm robotics.

### 5.2 Model-to-model transformation from SML to ARGoS XML files

ARGoS is a multi-engine simulator for swarm robotics. ARGoS has two main components that need to be defined in order to specify a mission: the XML configuration file and the so-called loop functions. Using the ARGoS XML file, users can specify the simulated space. ARGoS provides a way to specify several entity types. Each entity type stores information about a specific aspect of the simulation. It includes the position and the orientation of each object in the environment such as obstacles, light sources, boxes, and robots. The file is highly customizable and extendable—new entity types can be easily added and new features of the entities can be easily adapted and adjusted.

The loop functions are user-defined functions that are executed in strategic points of the simulation loop. Developers can customize the initialization and the end of an experiment, and add custom functions to be executed before and/or after each simulation step. Loop functions allow one to access and modify the entire state of the simulation. In particular, loop functions are a convenient way for computing relevant performance metrics used to measure the success of a mission.

**Figure 7.**
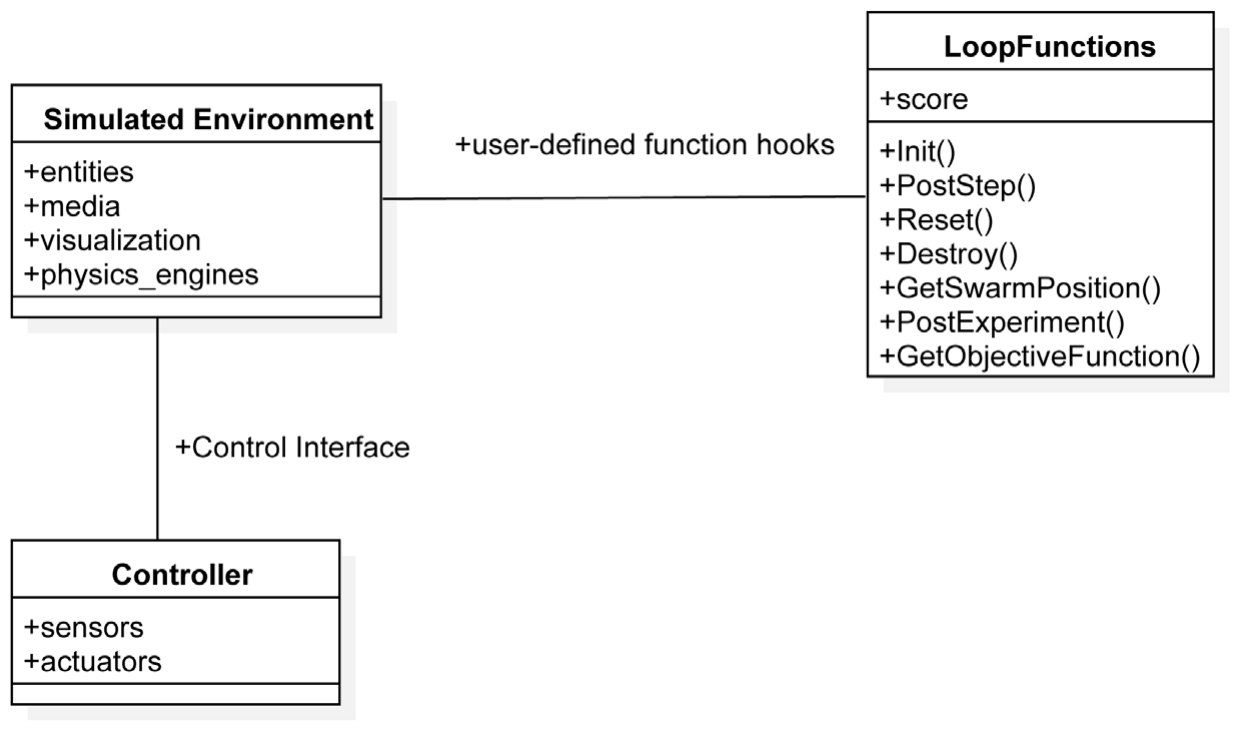
ARGoS architectural model.

Using model-to-model transformation techniques from the mission specification defined in SML, we automatically generate the XML file that is used by ARGoS to describe the simulation space and the loop functions that are used by ARGoS to run experiments. The mission models that are used by ARGoS are represented in Figure 7.

The Simulated Environment and the Controller are specified in the .xml file. A fragment of the generated XML configuration file is shown in Listing 4. It consists of five parts: experiment configuration details, definition of loop functions, specification of controllers, specification of the environment (the arena) and e-puck specification and distribution in the environment.

**Listing 4.**
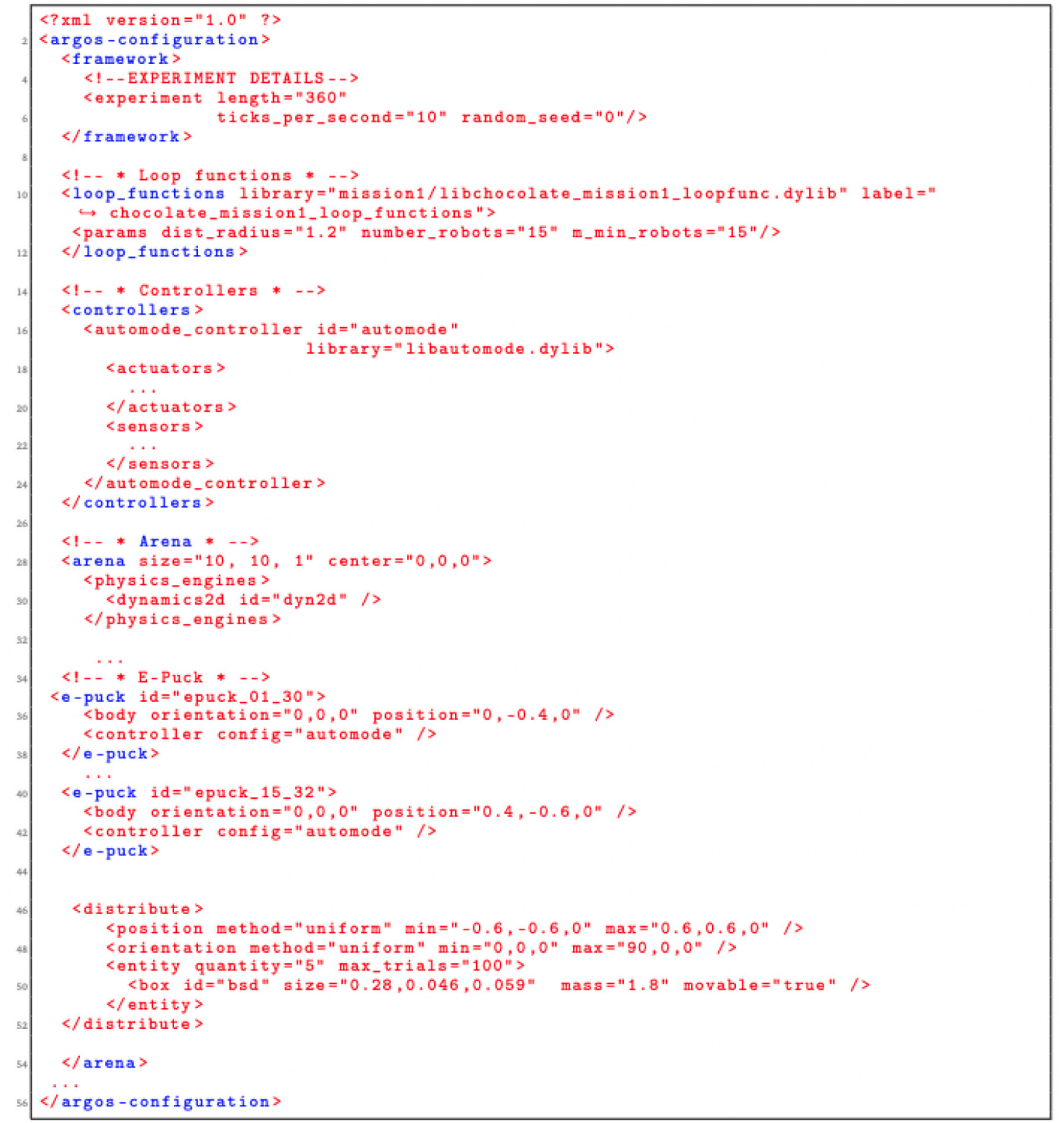
A fragment of the generated XML configuration file for Aggregation.

The loop functions are defined in a C++ file. We describe the main loop functions that are generated from the SML specification (fragment of the generated C++ file is shown in Listing 5). Init is a function that is used to instantiate all mission elements. We use it to create all mission entities, including the robots and the environment elements. PostStep is a function that is executed after each simulation steps. In this function, we iterate through the robot swarms and perform calculations, based on the definition of the objective function. In our scenarios, we used the concepts of reward and penalty to increase or decrease the value of the variable score which is an information on how well the robot swarm is performing the mission. GetSwarmPosition() is a function that defines the region where the robots are placed at the start of the mission. Here, we position each of the robots in the environment using some of the preexisting algorithms for placement. We only specify the boundaries of the region that is used for initial placement. Reset() is a function that is used by Chocolate to reset all variables before initializing a new run. PostExperiment() is a function that is executed after the mission is over. In our case, this function reports only the value of the ObjectiveFunction. GetObjectiveFunction() is a function that is used to return the score of an experimental run for a mission. If the score is high, Chocolate considers that the robot swarm performs the mission well.

**Listing 5.**
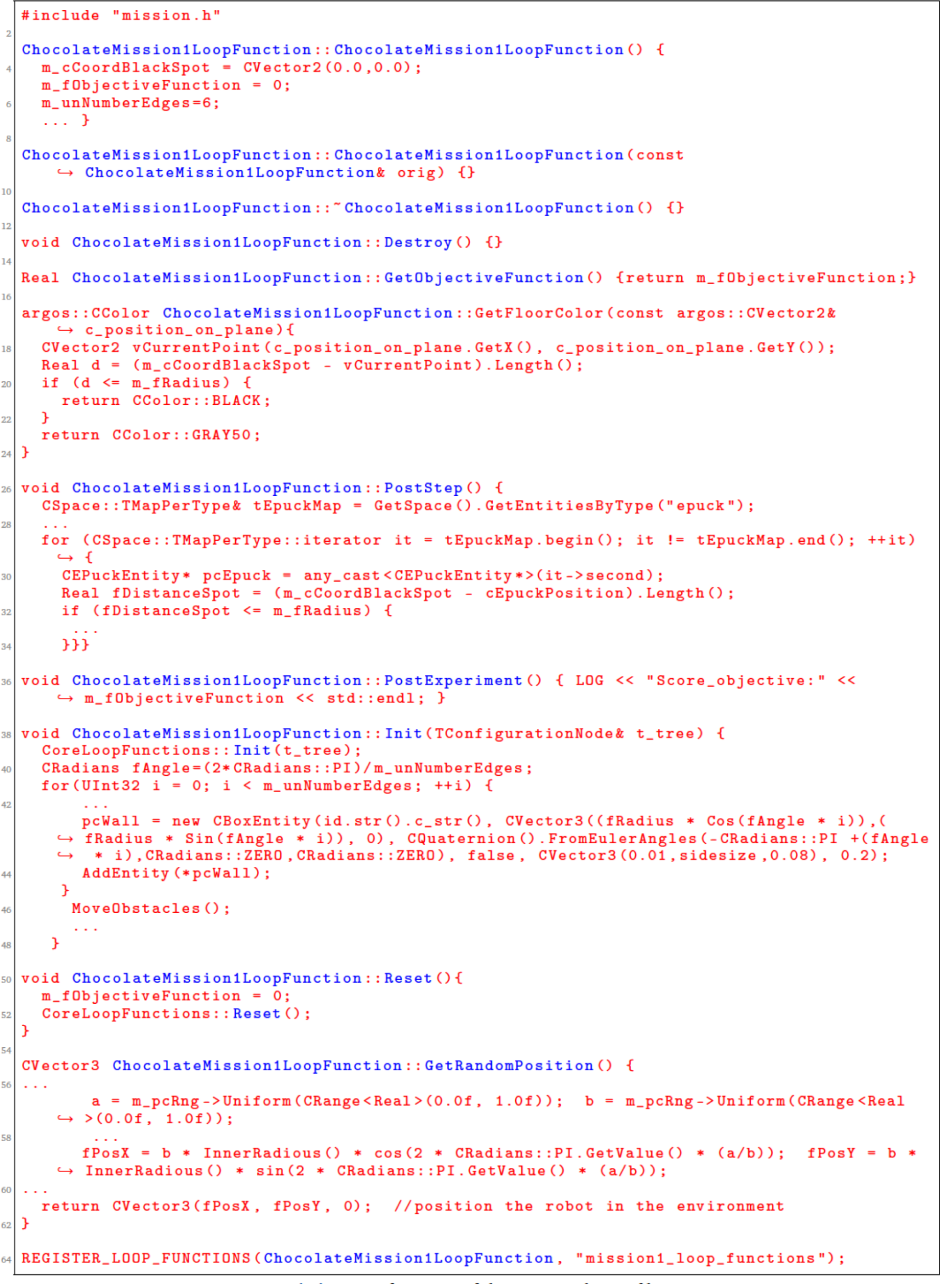
A fragment of the generated C++ file.

### 5.3 Validation of the SML implementation

The internal consistency of SML is critical for a fully integrated design process. To validate the internal consistency of the language we followed these steps:

1. **Instantiate a mission that contains all language constructs.** We created an instance of a mission that contains (almost) all language constructs. Furthermore, we specified smaller instances that instantiate the elements that could not be included in a single large instance due to exclusion constraints in the language.
2. **Validate the language in Xtext**. We created a set of validation rules that each mission instance should comply to. First, we created validation rules that ensure the structure of the mission instance. Each instance of a mission specification must contain a specification of the arena, specification of the environment, specification of the robot swarm and the objective that is measured. If any of these elements are missing, an error to the user is reported. Second, each physical element must be fully specified in terms of its location. The robot swarm, obstacles, lights, patches and all other environment elements must be located in a region of the arena. Third, all environment elements in the environment must be unambiguously specified in terms of their dimensions. As SML supports different type of specification variations (through a set of vertices, through side size, through a combination of a side size and a size of a diagonal), we created rules that verify that each specification is unambiguous representation of a physical element i.e. does not have any missing information (e.g., a designer that specifies a circle with a radius, must also specify the center of the circle, otherwise the information is incomplete.) These rules help the designer to double check semantic inconsistencies between different environmental elements.
3. **Check consistency of the generated model files.** To validate the consistency of the generated files, we performed the following analysis:
  • XML configuration consistency: For each system specification, we loaded the generated configuration file in ARGoS to confirm their internal consistency. Each configuration was successfully loaded by ARGoS. If there is an error in the configuration file, ARGoS is not able to load the file or if it loads it, some elements in the visualization will be missing.
  • Use the Chocolate design process to check for coherence and consistency of the loop functions. For each generated loop function file, we run Chocolate to confirm the validity. Chocolate is not able to run if the XML configuration is not properly specified or if the required functions Init(), PostStep(), GetSwarmPosition(), PostExperiment() are not instantiated ( Figure 7). If the loop functions contain a syntactical error, Chocolate throws an exception.

## 6 Demonstration

### Setup

To demonstrate the generality of SML, we present three different missions for which code was automatically generated and ported to real robots (see *Underlying data* ^53^). In the demonstration, we use e-puck robots ^21^ equipped with several extension boards ^54^, including the range-and-bearing board ^55^. We specify three missions in the SML editor. When we save a mission specification in the editor, the code generation process starts and generates an ARGoS .xml file and a .cpp file that contains the loop-functions that measure the performances of the swarm on the mission at hand. These files are used as artifacts for Chocolate ^1^. Chocolate generates control software for the reference model of the e-puck reported in Table 1 ^48^.

## Results

The following examples demonstrate the applicability of SML. For each of the three missions, Chocolate is executed 10 times to obtain 10 instances of control software. Each design process relies on a maximum of 200000 simulated runs. The simulator adopted in the study is ARGoS3, beta 48. We evaluated each instance of control software obtained by Chocolate in simulation. The best one was chosen and evaluated on real robots, as well.

In Listing 6, we specify *Aggregation on one spot*. First, we identify the shape of the arena, which is a *Hexagon* with sides of 1 m. Then, we declare various environmental elements and their location in a global coordinate system with an origin at the center of the arena. In this context, at line 4, we specify a circular black patch placed in the center of the arena with radius of 25 cm. At line 5, we specify the number and type of robots that will be used in the mission. We use 15 e-pucks distributed across the whole arena. At line 6, we define the details of the mission. Here, we explicitly state the mission goal and connect it to environmental elements. In this mission, the robots must aggregate on the black patch *c*1. At line 7, we specify that the mission completion time is 360 seconds.

**Listing 6.**
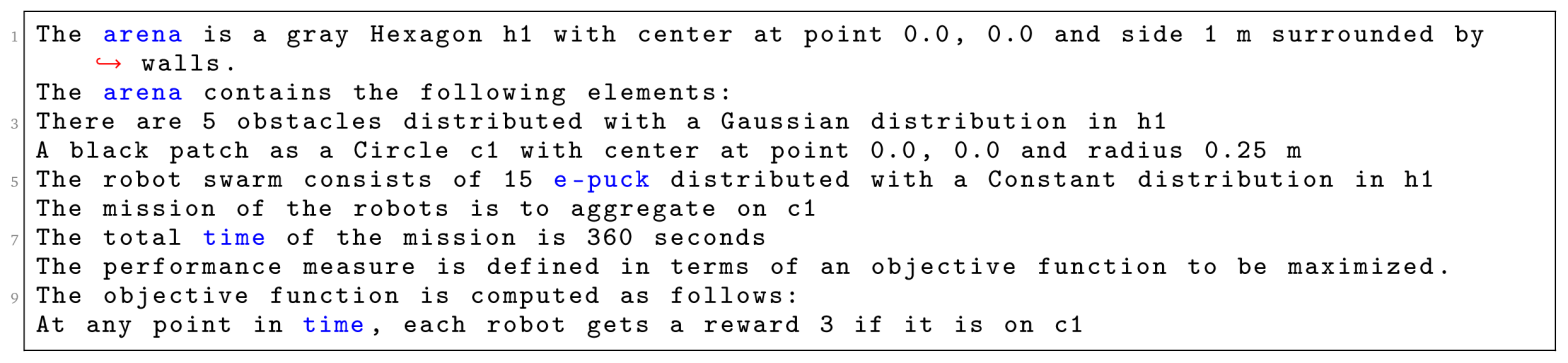
Mission specification.

**Figure 8.**
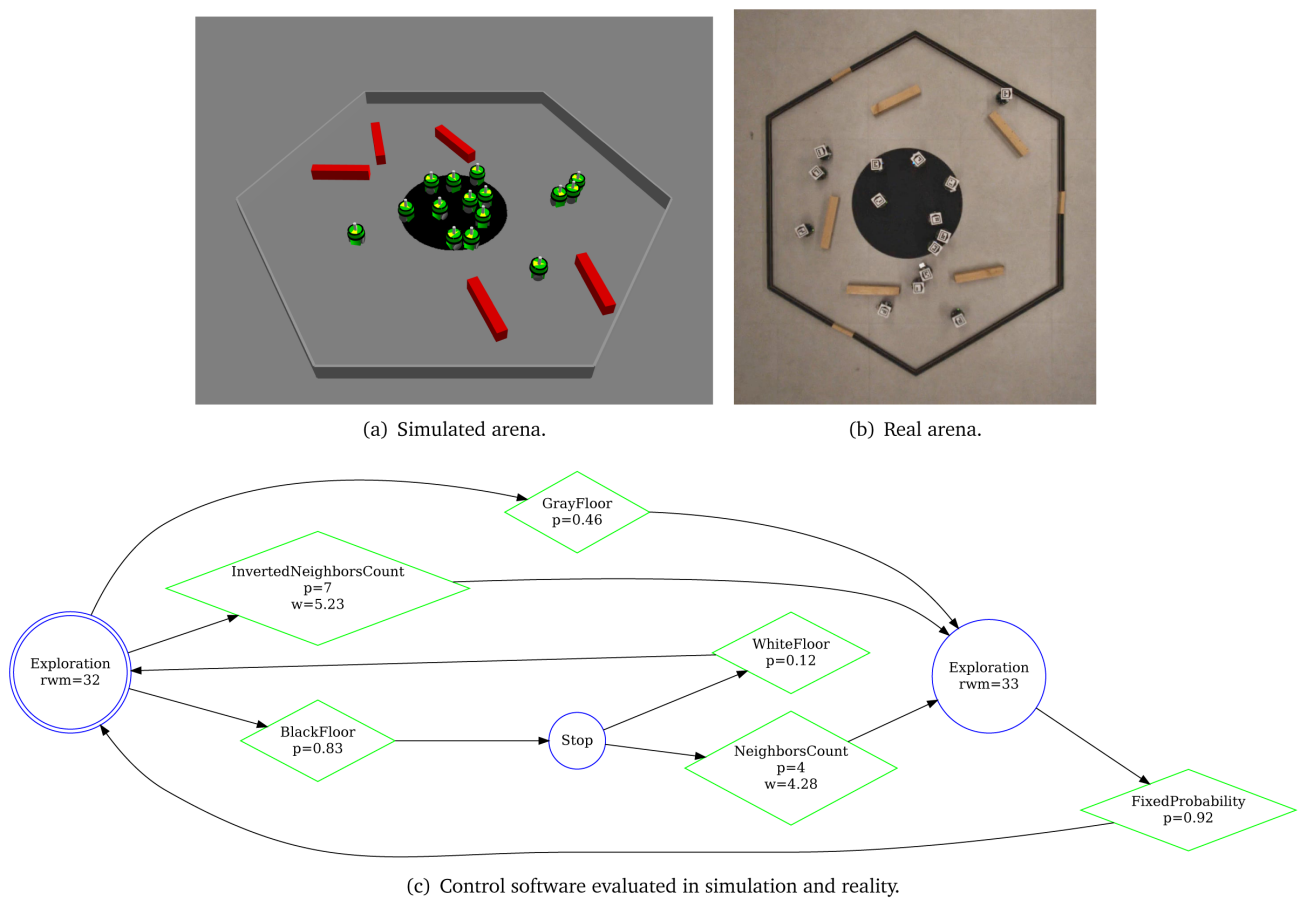
Aggregation on one spot.

At the end, we finish our specification with the definition of the objective function (line 9–10)—we state which concepts of the mission should be measured: we reward each robot that position itself on the black patch *c*1. Using the aforementioned mission specification, we generate the control software shown in Figure 8(c). The performance of the control software is evaluated in simulation— Figure 8(a) and on real robots— Figure 8(b).

**Figure 9.**
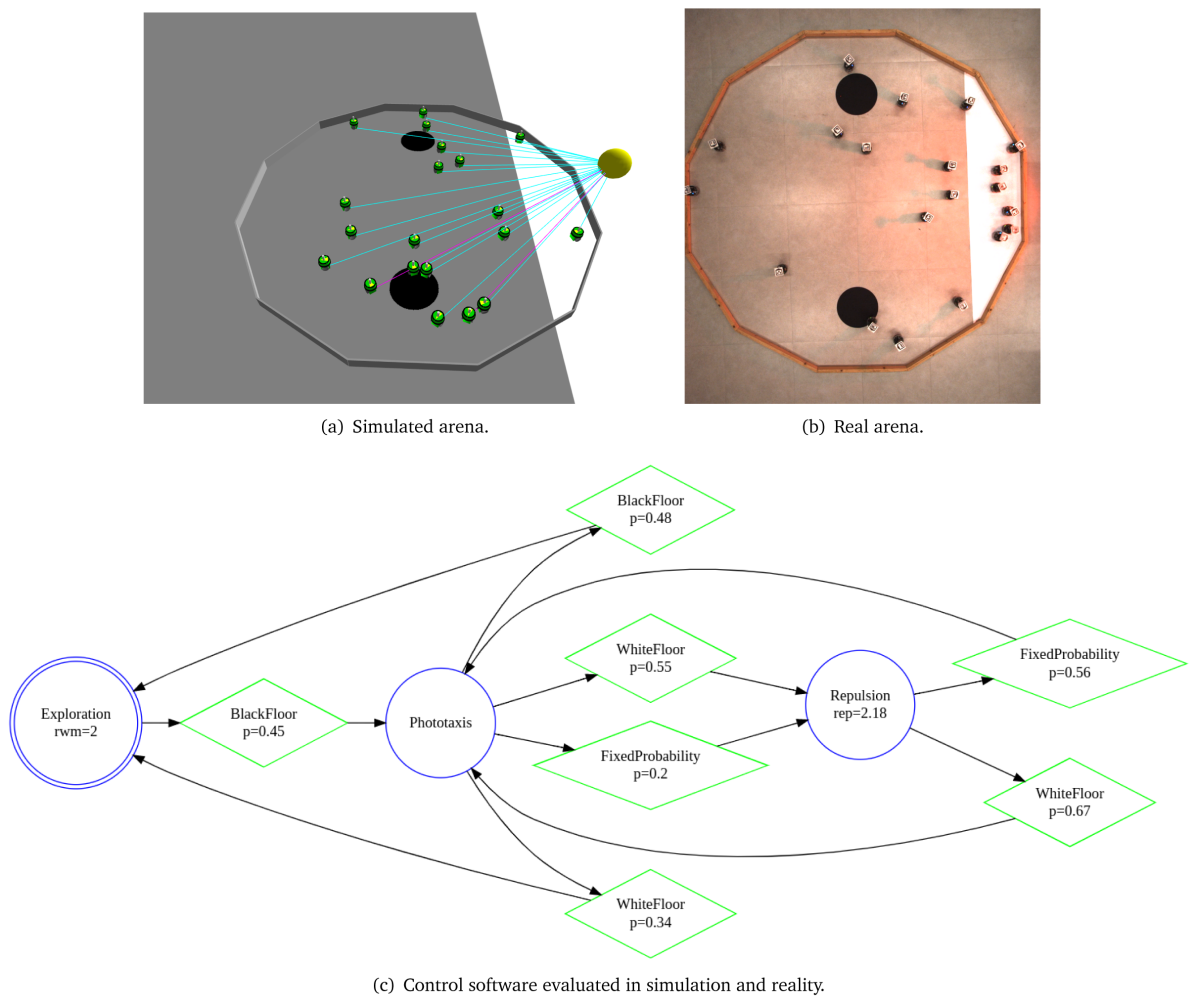
Foraging.

In Listing 7, we specify a *Foraging* mission with two sources and one nest. The arena is a dodecagon with a side of 0.66 m (line 1). We declare 4 environmental elements (line 2 – 6). For each environmental element, we specify its position in a global coordinate system with origin at the center of the arena. We declare a light source with intensity 5.0, two food sources located on *b*1 and *b*2 and a nest *w*1. At line 7, we state that 20 e-puck robots will be used in the mission. At line 8, we state the explicit details about the mission—connecting the mission goals (collect food, bring food) with their corresponding environmental elements (Circle b1, Circle b2, Region w1). At line 9, we define that the mission completion time is 270 seconds. At the end, we declare the specific elements of the objective function—we quantify when an individual robot arrives in one of the black patches *b*1 or *b*2 and gets back to its nest *w*1. Using the aforementioned mission specification, we generate the control software shown in Figure 9(c). The performance of the control software is evaluated in simulation— Figure 9(a) and on real robots— Figure 9(b).

**Listing 7.**
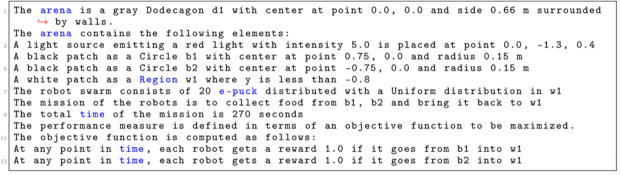
Mission specification.

**Figure 10.**
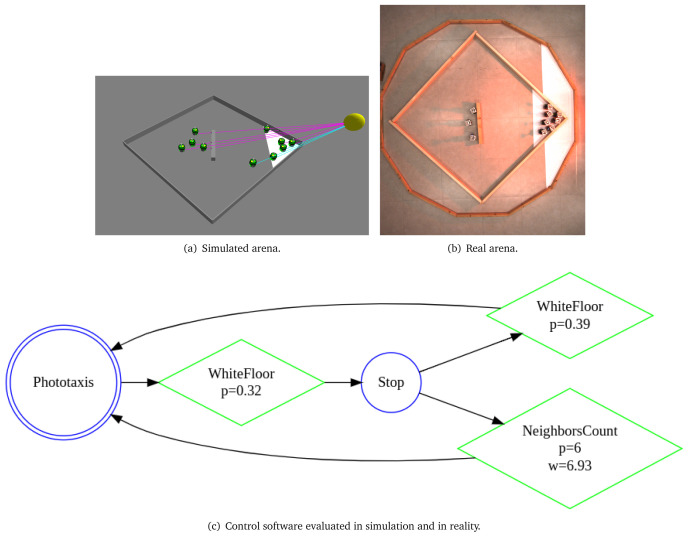
Migration with an obstacle.

In Listing 8, we specify *Migration with an obstacle*. The arena of the this mission is a square with a side of 1.5 m (line 1). We declare four environmental elements (line 2 - 6). For each environmental element, we specify its position in a global coordinate system with origin at the center of the arena. We declare a light source with intensity 3.0, a triangular white patch *t*1—an area to which the swarm must move, a large obstacle *r*_0_ *b* in the center of the arena and initial location of the swarm *r t*. At line 7, we state that 10 e-puck robots will be used in the mission. At line 8, we state the explicit details about the mission—connecting the mission goals (migration to an area) with their corresponding environmental elements (Triangle t1). At line 9, we define that the mission completion time is 300 seconds. In the end, we declare the specific elements of the objective function (line 9–13): we reward the swarm if more than 5 robots are at the target location. Moreover, every time the swarm receives a reward, we add a small penalty for each robot outside the target area. Using the aforementioned mission specification, we generate the control software shown in Figure 10(c). The performance of the control software is evaluated in simulation— Figure 10(a) and on real robots— Figure 10(b).

**Listing 8.**
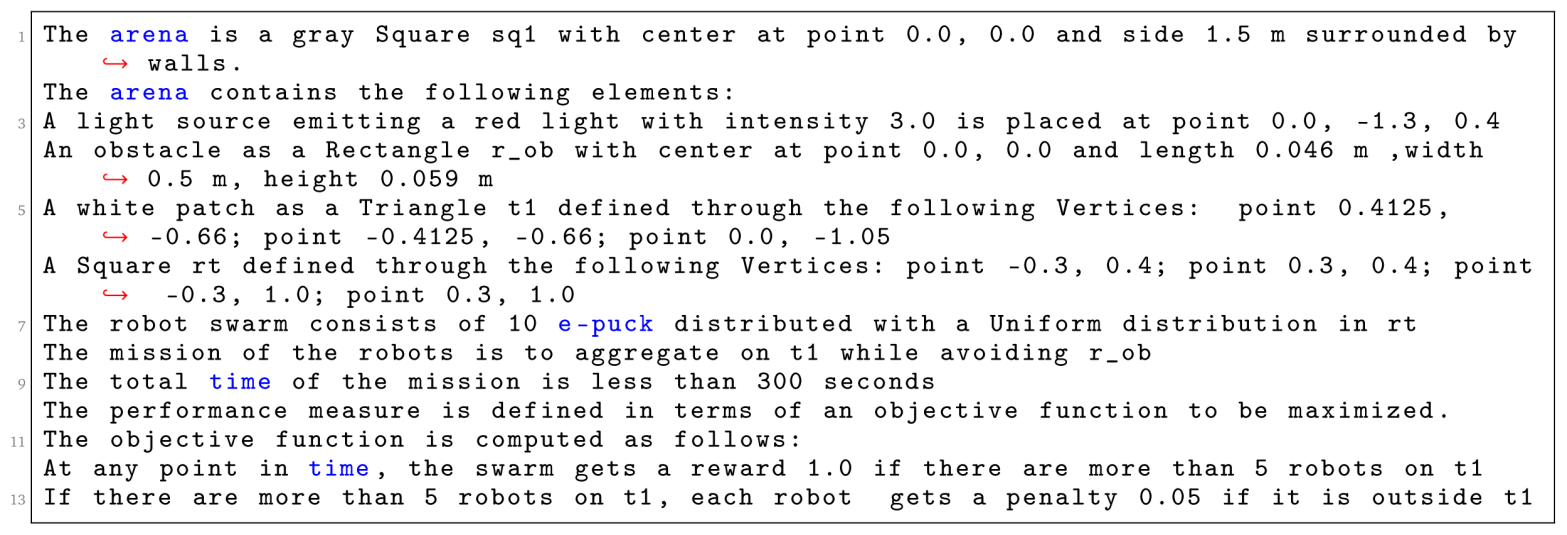
Mission specification.

The generated code, the data collected in simulation and in reality, and the videos of the behavior by the swarm of physical robots are available online as supplementary material ^53, 56^.

## 7 Conclusions

We presented a first instance of a fully automatic and integrated process for the design of collective behaviors for robot swarms. The novel contribution in this paper is the definition of a specification language and an automatic approach that transforms a formal mission specification into a configuration setup needed to run the design by optimization of control software using Chocolate. We introduced SML, a textual language to specify a mission that can be accomplished by a robot swarm. From the mission specification, we automatically generated code and deployed it on real robots. We demonstrated the applicability of SML on three missions.

The current implementation of SML supports the specification of missions in which rewards and penalties can be expressed with reference to regions: depending on whether robots or objects are in a particular part of the environment at a given moment in time. Missions like aggregation, foraging and collective exploration can be modeled through the concept of a region. However, collective decision making or coordinate navigation missions discussed in Section 4.1 (e.g., flocking, consensus achievement, and task allocation) cannot be modeled using the current implementation of SML.

Future work will develop along three lines. First, we will work on the language extensibility. We plan to extend the set of constructs to support the specification of new classes of missions. We will introduce new indicators and patterns to measure success of new mission types. Moreover, we plan to enrich the language by introducing quality attributes as part of the mission specification. Safety, performance, and energy-efficiency are just a few important quality attributes that should be modeled as separate language constructs in the definition of missions for robot swarms. Introducing quality attributes in the mission specification process will contribute towards a better definition of mission objectives. Moreover, we will work on a formal language validation. After employing new constructs that should support new classes of missions, we plan to perform model validation to understand if there are constructs that are not covered by the current implementation of SML. We plan to create a mission generator and generate a set of mission instances that can help us to analyze the coverage of the language. This will help us to increase the number of mission classes that can be captured by the language implementation.

Second, we plan on developing our contribution towards a systematic methodology for designing robot swarms. We are focused on a full-fledged linear automatic design process as we were making our first steps in closing all the gaps in the fully automatic design process: a swarm designer is able to specify robot swarm requirements, but also include details about the design setup to be used to obtain the desired robot swarm. This includes information on which automatic design method use, how many simulation runs to perform, and information on the target environment. In this contribution, these details were manually specified as part of the automatic design method (we considered only Chocolate and a fixed design budget of 200K simulation runs). Moreover, to obtain a fully systematic integrated design process, swarm designers need to be able to select a set of predefined individual behaviours that can be used by the automatic design method to generate the control software. In this work, six behaviours were considered and they were selected as part of the automatic design method. Swarm designers need to be able to specify these details in the early phases of the design process, together with the definition of the system requirements. After obtaining a full-fledged linear automatic design process, we plan to move to an iterative design process where there is a feedback loop between design and testing before deployment of the control software in the target environment.

Third, we will focus on the usability of our integrated design process by investigating two research directions: i) We will perform an extensive user study to investigate SML’s usability. To obtain deeper insights on how easily swarm designers can use SML for swarm missions, we will define a rigour protocol for evaluation based on objective qualitative and quantitative metrics that should demonstrate the usefulness of our domain-specific language; ii) We will develop a graphical user interface for non-expert users. The aim of the graphical interface is defining a process for non-expert users that is user friendly and less prone to input errors.

## Data Availability

Zenodo: Integrated automatic design process for robot swarms. https://zenodo.org/record/5184720 ^53^. This project contains the following underlying data: SML (Swarm Mission Language) related files: (i) specification files used to create missions; (ii) generated files to be used by an optimization method AutoMoDe related files: (i) the log files running AutoMoDe - an optimization method that generates control software for different missions; (ii) generated control software Demonstration: (i) snapshots and videos of running the missions on real robots Data are available under the terms of the Creative Commons Attribution 4.0 International license (CC-BY 4.0).

## Software availability

**ARGoS3-AutoMoDe** for the implementation of AutoMoDe-Chocolate

- Source code available from: https://github.com/demiurge-project/ARGoS3-AutoMoDe
- Archived source code at time of publication: https://doi.org/10.5281/zenodo.4849541;
- License: MIT license.

**Demiurge-epuck-dao** for the reference model of the robots used by the AutoMoDe design method

- Source code available from: https://github.com/demiurge-project/demiurge-epuck-dao
- Archived source code at time of publication: https://doi.org/10.5281/zenodo.4849535
- License: MIT license.

**Argos3-epuck** for the e-puck robot ARGoS3 plugin

- Source code available from: https://github.com/demiurge-project/argos3-epuck
- Archived source code at time of publication: https://doi.org/10.5281/zenodo.4882714
- License: MIT license.

**ARGoS3** for the ARGoS3 simulator

- Source code available from: https://github.com/ilpincy/argos3
- Archived source code at time of publication: https://doi.org/10.5281/zenodo.4889111
- License: Creative Commons Attribution 4.0 International license (CC-BY 4.0).

**Irace** for the Iterated F-race algorithm

- Software available from: https://cran.r-project.org/package=irace
- Archived source code at time of publication: https://doi.org/10.5281/zenodo.4888996
- License: Creative Commons Attribution 4.0 International license (CC-BY 4.0).

## References

[1] Francesca G Brambilla M Brutschy A : AutoMoDe-Chocolate: automatic design of control software for robot swarms. *Swarm Intell.* 2015;9(2/3):125–152. 10.1007/s11721-015-0107-9

[2] Dorigo M Birattari M Brambilla M : Swarm robotics. *Scholarpedia.* 2014;9(1):1463. 10.4249/scholarpedia.1463

[3] Bozhinoski D Birattari M : Designing control software for robot swarms: Software engineering for the development of automatic design methods. In: *ACM/IEEE 1st International Workshop on Robotics Software Engineering RoSE.*New York, ACM,2018;33–35. Reference Source

[4] Brambilla M Ferrante E Birattari M : Swarm robotics: a review from the swarm engineering perspective. *Swarm Intell.* 2013;7(1):1–41. 10.1007/s11721-012-0075-2

[5] Quinn M Smith L Mayley G : Evolving controllers for a homogeneous system of physical robots: structured cooperation with minimal sensors. *Philos Trans A Math Phys Eng Sci.* 2003;361(1811):2321–43. 10.1098/rsta.2003.1258 14599322

[6] Trianni V : Evolutionary swarm robotics: evolving self-organising behaviours in groups of autonomous robots. Springer,2008;108. 10.1007/978-3-540-77612-3

[7] Francesca G Brambilla M Brutschy A : AutoMoDe: a novel approach to the automatic design of control software for robot swarms. *Swarm Intell.* 2014;8(2):89–112. 10.1007/s11721-014-0092-4

[8] Birattari M Ligot A Hasselmann K : Disentangling automatic and semi-automatic approaches to the optimization-based design of control software for robot swarms. *Nat Mach Intell.* 2020;2(9):494–499. 10.1038/s42256-020-0215-0

[9] Brugali D Prassler E : Software engineering for robotics. *IEEE Robot Autom Mag.* 2009;16(1):9–15. 10.1109/MRA.2009.932127

[10] Di Ruscio D Malavolta I Pelliccione P : A family of domain-specific languages for specifying civilian missions of multi-robot systems. In: *First Workshop on Model-Driven Robot Software Engineering-MORSE*. York, UK,2014;13–26. Reference Source

[11] Bozhinoski D Di Ruscio D Malavolta I : Flyaq: Enabling non-expert users to specify and generate missions of autonomous multicopters. In: *2015 30th IEEE/ACM International Conference on Automated Software Engineering (ASE)*. San Diego, CA, USA, IEEE,2015;801–806. 10.1109/ASE.2015.104

[12] Bozhinoski D Garlan D Malavolta I : Managing safety and mission completion via collective run-time adaptation. *J Syst Archit.* 2019;95:19–35. 10.1016/j.sysarc.2019.02.018

[13] Hoos HH : Programming by optimization. *Commun ACM.* 2012;55(2):70–80. 10.1145/2076450.2076469

[14] Francesca G Birattari M : Automatic design of robot swarms: achievements and challenges. *Front Robot AI.* 2016;3(29):1–9. 10.3389/frobt.2016.00029

[15] Birattari M Ligot A Bozhinoski D : Automatic off-line design of robot swarms: a manifesto. *Front Robot AI.* 2019;6:59. 10.3389/frobt.2019.00059 33501074 PMC7806002

[16] Schmidt DC : Model-driven engineering. *IEEE Computer.* 2006;39(2):25. Reference Source

[17] Schlegel C Lotz A Lutz M : Model-driven software systems engineering in robotics: covering the complete life-cycle of a robot. *Info Technol.* 2015;57(2):85–98. 10.1515/itit-2014-1069

[18] Schlegel C Haßler T Lotz A : Robotic software systems: From code-driven to model-driven designs. In: *2009 International Conference on Advanced Robotics*. Munich, Germany, IEEE,2009;1–8. Reference Source

[19] Hasselmann K Ligot A Ruddick J : Empirical assessment and comparison of neuro-evolutionary methods for the automatic off-line design of robot swarms. *Nat Commun.* 2021;12(1):4345. 10.1038/s41467-021-24642-3 34272382 PMC8285396

[20] Pinciroli C Trianni V O’Grady R : ARGoS: a modular, parallel, multi-engine simulator for multi-robot systems. *Swarm Intell.* 2012;6(4):271–295. 10.1007/s11721-012-0072-5

[21] Mondada F Bonani M Raemy X : The e-puck, a robot designed for education in engineering. In P. Gonçalves, P. Torres, and C. Alves, editors, *Proceedings of the 9th Conference on Autonomous Robot Systems and Competitions*. Portugal, Instituto Politécnico de Castelo Branco,2009;59–65. Reference Source

[22] Nolfi S Floreano D Floreano DD : Evolutionary robotics: The biology, intelligence, and technology of self-organizing machines. MIT press,2000. Reference Source

[23] Nolfi S : Behavioral and Cognitive Robotics: An Adaptive Perspective. Institute of Cognitive Sciences and Technologies, National Research Council, CNR-ISTC, Roma, Italy,2021. Reference Source 10.1007/s10339-011-0402-3 21468745

[24] Hauert S Zufferey JC Floreano D : Reverse-engineering of artificially evolved controllers for swarms of robots. In: *2009 IEEE Congress on Evolutionary Computation.*IEEE,2009;55–61. 10.1109/CEC.2009.4982930

[25] Ligot A Birattari M : Simulation-only experiments to mimic the effects of the reality gap in the automatic design of robot swarms. *Swarm Intell.* 2020;14(1):1–24. 10.1007/s11721-019-00175-w

[26] Koos S Mouret JB Doncieux S : The transferability approach: crossing the reality gap in evolutionary robotics. *IEEE Trans Evol Comput.* 2013;17(1):122–145. 10.1109/TEVC.2012.2185849

[27] Haasdijk E Bredeche N Nolfi S : Evolutionary robotics. *Evol Intell.* 2014;7:69–70. 10.1007/s12065-014-0113-7

[28] Birattari M Ligot A Francesca G : AutoMoDe: a modular approach to the automatic off-line design and fine-tuning of control software for robot swarms. In: Nelishia Pillay and Rong Qu, editors, *Automated Design of Machine Learning and Search Algorithms*. Nature, Cham, Switzerland,2021;73–90. 10.1007/978-3-030-72069-8_5

[29] Geman S Bienenstock E Doursat R : Neural networks and the bias/variance dilemma. *Neural Comput.* 1992;4:1–58. Reference Source

[30] Birattari M Stützle T Paquete L : A racing algorithm for configuring metaheuristics. In: W.B. Langdon and *et al*., editors, *Proceedings of the Genetic and Evolutionary Computation Conference, GECCO*. San Francisco CA Morgan Kaufmann.2002;11–18. Reference Source

[31] Birattari M : Tuning Metaheuristics: A Machine Learning Perspective. Springer, Berlin, Germany,2009. 10.1007/978-3-642-00483-4

[32] Birattari M Yuan Z Balaprakash P : F-race and iterated f-race: An overview. In: Thomas Bartz- Beielstein, Marco Chiarandini, Luís Paquete, and Mike Preuss, editors, *Experimental Methods for the Analysis of Optimization Algorithms*. Springer, Berlin, Germany,2010;311–336. 10.1007/978-3-642-02538-9_13

[33] Franzin A Stützle T : Revisiting simulated annealing: A component-based analysis. *Comput Oper Res.* 2019;104:191–206. 10.1016/j.cor.2018.12.015

[34] Hasselmann K Birattari M : Modular automatic design of collective behaviors for robots endowed with local communication capabilities. *PeerJ Comput Sci.* 2020;6:e291. 10.7717/peerj-cs.291 33816942 PMC7924432

[35] Ligot A Kuckling J Bozhinoski D : Automatic modular design of robot swarms using behavior trees as a control architecture. *PeerJ Comput Sci.* 2020;6:e314. 10.7717/peerj-cs.314 33816965 PMC7924474

[36] Nordmann A Hochgeschwender N Wigand D : A survey on domain-specific modeling and languages in robotics.2016. Reference Source

[37] Neill CJ Laplante PA : Requirements engineering: the state of the practice. *IEEE Softw.* 2003;20(6):40–45. 10.1109/MS.2003.1241365

[38] Van Lamsweerde A : Goal-oriented requirements enginering: a roundtrip from research to practice. In: *Proc IEEE Int Requir Eng Conf, 2004*. Washington, DC USA, IEEE.2004;4–7. 10.1109/ICRE.2004.1335648

[39] Pinciroli C Beltrame G : Buzz: An extensible programming language for heterogeneous swarm robotics. In: *2016 IEEE/RSJ International Conference on Intelligent Robots and Systems (IROS)*. IEEE,2016;3794–3800. 10.1109/IROS.2016.7759558

[40] Beltrame G Merlo E Panerati J : Engineering safety in swarm robotics. In: *Proceedings of the 1st International Workshop on Robotics Software Engineering*.2018;36–39. 10.1145/3196558.3196565

[41] Brambilla M Brutschy A Dorigo M : Property-driven design for swarm robotics: A design method based on prescriptive modeling and model checking. *ACM Transactions on Autonomous and Adaptive Systems.* 2015;9(4):17. 10.1145/2700318

[42] Kelly S Tolvanen JP : Domain-specific modeling: enabling full code generation. John Wiley & Sons,2008. 10.1002/9780470249260

[43] Dixon C Winfield AFT Fisher M : Towards temporal verification of swarm robotic systems. * Rob Auton Syst.* 2012;60(11):1429–1441. 10.1016/j.robot.2012.03.003

[44] Bonani M Longchamp V Magnenat S : The marxbot, a miniature mobile robot opening new perspectives for the collective-robotic research. In: *2010 IEEE/RSJ International Conference on Intelligent Robots and Systems*. Piscataway, NJ, IEEE.,2010;4187–4193. 10.1109/IROS.2010.5649153

[45] Riedo F Chevalier M Magnenat S : Thymio II a robot that grows wiser with children. In: *2013 IEEE workshop on advanced robotics and its social impacts*. Tokyo, Japan, IEEE.2013;187–193. 10.1109/ARSO.2013.6705527

[46] Rubenstein M Ahler C Nagpal R : Kilobot: A low cost scalable robot system for collective behaviors. In: *2012 IEEE Int Conf Robot Autom*. St Paul, MNUSA, IEEE.,2012;3293–3298. 10.1109/ICRA.2012.6224638

[47] Soares JM Navarro I Martinoli A : The Khepera IV mobile robot: performance evaluation, sensory data and software toolbox. In *Robot 2015: Second Iberian Robotics Conference*. Lisbon, Portugal, Springer.2016;767–781. 10.1007/2F978-3-319-27146-0_59

[48] Hasselmann K Ligot A Francesca G : Reference models for AutoMoDe. Technical Report TR/IRIDIA/2018-002, IRIDIA, Université libre de Bruxelles, Belgium,2018. Reference Source

[49] Dwyer MB Avrunin GS Corbett JC : Patterns in property specifications for finite-state verification. In: *Proceedings of the 1999 International Conference on Software Engineering (IEEE Cat. No. 99CB37002)*. Los Angeles, CA, USA, IEEE.,1999;411–420. 10.1145/302405.302672

[50] Autili M Grunske L Lumpe M : Aligning qualitative, real-time, and Probabilistic property specification patterns using a structured english grammar. *IEEE Trans Softw Eng.* 2015;41(7):620–638. 10.1109/TSE.2015.2398877

[51] Eysholdt M Behrens H : Xtext: implement your language faster than the quick and dirty way. In: *Proceedings of the ACM International Conference Companion on Object Oriented Programming Systems Languages and Applications Companion*. Nevada, USA, ACM.2010;307–309. 10.1145/1869542.1869625

[52] Mondada F Guignard A Bonani M : Swarm-bot: from concept to implementation. In: *IEEE/RSJ International Conference on Intelligent Robots and Systems*. Piscataway, NJ, IEEE.2003;2:1626–1631. 10.1109/IROS.2003.1248877

[53] Bozhinoski D Birattari M : Integrated automatic design process for robot swarms.2021. 10.5281/zenodo.5184720 PMC10446085 37645125

[54] Garattoni L Francesca G Brutschy A : Software infrastructure for e-puck (and TAM). Technical Report TR/IRIDIA/2015-004, IRIDIA, Université libre de Bruxelles, Belgium,2015. Reference Source

[55] Gutiérrez I Campo A Dorigo M : Open e-puck range & bearing miniaturized board for local communication in swarm robotics. In: Kinugawa Kosuge, editor, *IEEE Int Conf Robot Autom*.ICRA. Piscataway, NJ, IEEE.2009;3111–3116. 10.1109/ROBOT.2009.5152456

[56] Bozhinoski D Birattari M : Requirements specification for swarm robotics: Supplementary material.2020. Reference Source

