# Supplementary material for: Towards an integrated automatic design process for robot swarms
Source: Open Res Eur. 2022 Nov 4;1:112. Originally published 2021 Sep 27. [Version 2] doi: 10.12688/openreseurope.14025.2 (PMC10446085; doi:10.12688/openreseurope.14025.2)
Supplement: Peer review history

# Peer review: Reviewer response for version 2

**DOI:** 10.21956/openreseurope.16243.r30379

**Published:** 2022-11-04

**Authors:** Edmund Hunt, James Ward

**Affiliations:** 1 University of Bristol, Bristol, UK

I have no further comments to make.

Is the study design appropriate and does the work have academic merit?

Yes

Is the work clearly and accurately presented and does it cite the current literature?

Partly

If applicable, is the statistical analysis and its interpretation appropriate?

Not applicable

Are all the source data underlying the results available to ensure full reproducibility?

Yes

Are the conclusions drawn adequately supported by the results?

Partly

Are sufficient details of methods and analysis provided to allow replication by others?

Yes

Reviewer Expertise:

Swarm robotics

We confirm that we have read this submission and believe that we have an appropriate level of expertise to confirm that it is of an acceptable scientific standard.

# Peer review: Reviewer response for version 1

**DOI:** 10.21956/openreseurope.15113.r29339

**Published:** 2022-06-07

**Authors:** Edmund Hunt, James Ward

**Affiliations:** 1 University of Bristol, Bristol, UK

This paper presents work on a textual language (‘SML’) for specification of swarm robot missions, and an engine to transform this SML into a form that can be used by the previously published ‘AutoMoDe-Chocolate’ automatic controller designer. The pipeline from mission specification to deployment is demonstrated in three example missions.

 The ambitious goal to show a ‘fully integrated design process’ for swarm deployment, using off-line optimization methods, is met with an initial proof concept. Obviously, much work remains to be done on showing that this approach will be successful in ‘real world’ environments, both in relation to mission success (relative to alternative approaches, e.g. hand-design of controllers) and usability for non-expert users. But it is a useful step in that direction, and the paper therefore is a meaningful contribution to the field.

 Specific points arising:

- Given the focus on swarms and emergent behaviour – e.g. the Introduction “Hence, the collective behavior of a robot swarm is a result of the local interactions between the individual robot and its neighbors and its environment” – the three missions tested do not especially rely on neighbour interactions/emergence, and could equally be tested on a single robot? And so the real-world validation is arguably on the boundary of what could properly be called swarm robotics, and I look forward to the promised future work on e.g. collective decision-making missions.
- In the Abstract, I would contest the claim that ‘swarm designers need to follow standardised automatic design processes…’ I suggest ‘may benefit from following’ rather than ‘need to’.
- In section 3.1, it is claimed that the ‘approach…enables…non-expert users (non-technical end-users) to specify requirements for robot swarms…’. Realistically though, how accessible is SML to a non-expert? Would some kind of graphical interface be easier to use and less prone to input errors?
- In section 3.2, the design budget is 200k simulation runs. This seems like a lot. What is the run time on what hardware? Why is such a large number necessary? What would be possible with e.g. 2k runs?
- ‘Chocolate’ operates on only six low-level behaviours and six conditions. This is fine for lab-based swarm concepts, but how extensible is this framework into the ‘real world’, e.g. with ROS-based robots running with sensors such as LiDARs, depth cameras, etc – robots with greater ‘spatial intelligence’ to understand context, performing more complex missions? I wonder if BDRML is a relevant approach. ^1^
- It would be interesting to test missions where the simulation is not quite matching the deployed environment; e.g. with the aggregation mission, would it make much difference to specify 3 obstacles rather than the actual 5?
- On Fig. 1, realistically for real-world deployments, will there be a feedback loop between step 2 and 3? Take driverless cars as an example, development requires thousands of hours of trials and controller refinement. Are you focused on missions where iterative testing and design will not be possible? Do you have specific contexts in mind?
- I find the large ‘figures’ showing code to be better suited to supplementary information, it interrupts the paper flow and makes the page count overlong.
- Minor points: p.3 ‘alea’ – I am not familiar with this term, please clarify your meaning. p.3 ‘through the concept of region’ – awkward phrasing.

Is the study design appropriate and does the work have academic merit?

Yes

Is the work clearly and accurately presented and does it cite the current literature?

Partly

If applicable, is the statistical analysis and its interpretation appropriate?

Not applicable

Are all the source data underlying the results available to ensure full reproducibility?

Yes

Are the conclusions drawn adequately supported by the results?

Partly

Are sufficient details of methods and analysis provided to allow replication by others?

Yes

Reviewer Expertise:

Swarm robotics

We confirm that we have read this submission and believe that we have an appropriate level of expertise to confirm that it is of an acceptable scientific standard.

## References

[1] : Behaviour-Data Relations Modelling Language For Multi-Robot Control Algorithms. *2017 IEEE/RSJ International Conference on Intelligent Robots and Systems (IROS)* .2017; 10.1109/IROS.2017.8202231 727-732 10.1109/IROS.2017.8202231

# Response

**Published:** 2022-08-04

**Authors:** Darko Bozhinoski

**Affiliations:** 151927, The Netherlands

We are glad that the reviewer appreciated the work we have performed. In the following, we address his comments point by point.

- Concerning the idea of creating a graphical interface to facilitate the usage of SML, we extended the Conclusions by adding this research direction as a future contribution.
- Concerning the large design budget of 200k simulation runs, we would like to stress that the decision of adopting such a large design budget  is outside of the specific scope of the contribution we are making with this paper. We decided to adopt a large design budget, so that the design process has sufficient resources to obtain a controller that performs well. The focus here is on the specification of the mission and on the automatic process that transforms specifications into the input to be fed to Chocolate.
- Concerning the fact that the automatic design method Chocolate operates on only six low-level behaviours and 6 conditions, we would like to stress that the focus in this paper is not Chocolate per se, but rather how to define a fully automatic design process (from specifications to the actual execution of the mission). As we already mentioned in answer to the other reviewers, this work is only a first step towards an integrated automatic design process for robot swarms: a proof of concept implementation.  Extending the framework from a lab-based environment to a real-world environment is definitely an important issue that will be addressed in future research work. In the Behaviour-Data Relations Modelling Language (BDRML) [1] approach, the authors propose a methodology to represent robot behaviours, data, and a set of conditional relations between the different primitives. In contrast, the main focus in our work is on establishing an end-to-end automatic approach where from a mission specification in natural language, swarm control software can be obtained without focusing on the specificities of data structures and behaviours.
- Concerning the idea of testing the approach on missions where the simulation environment does not fully match the deployment environment, we would like to point out that our current approach already provides support for it. Many environmental features can be described in a probabilistic manner, meaning that the automatic design process generates control software that is trained on a representative set of environments that are different from the one into which the swarm is eventually deployed. We refer to a class of missions (environments) and we only make the working hypothesis that all the environments experienced in simulation and the real one into which the swarm is deployed are part of the same mission class.
- We agree with the reviewer’s comment that having a feedback loop in the automatic design process is an important part of the development of robust and reliable robot swarms. Yet, the setting we considered in the paper—that is, the linear life-cycle model—is the basic building block to achieve any more complex life-cycle. We feel that considering a lifecycle with a feedback loop will be an important future step. In this work, we focused on the simplest development model as we are making our first steps in closing the gaps in the fully automatic design process: starting from the specification of a mission, obtaining a swarm controller in simulation and finally deploying it in a real environment. We strongly believe that before we move to an iterative design process, we must focus on having a full-fledged linear automatic design process and fully master it.

We thank the reviewer for his valuable feedback.

   [1] Pitonakova L, Crowder R, Bullock S: Behaviour-Data Relations Modelling Language For Multi-Robot Control Algorithms. 2017 IEEE/RSJ International Conference on Intelligent Robots and Systems (IROS). 2017; 727-732 |

# Peer review: Reviewer response for version 1

**DOI:** 10.21956/openreseurope.15113.r28563

**Published:** 2022-05-30

**Authors:** Alan Millard

**Affiliations:** 1 Department of Computer Science, University of York, York, UK

This article presents a novel textual language for the specification of robot swarm missions called Swarm Mission Language (SML). Model-driven engineering techniques are used to automatically transform missions specified in SML into a format compatible with the previously published AutoMoDe-Chocolate optimisation-based design method. The authors demonstrate the potential of this integrated design process on three different example missions, with validation on physical robots.

 This work is a valuable contribution to the field, bridging the gap between the high-level specification of swarm behaviours and automated design methods that determine the behaviour of individual robots. Integration with an established simulation tool (ARGoS) and robot platform (e-puck) makes the approach accessible to the research community, and the source code and data have been made available to aid reproducibility.

 The current implementation of SML is relatively limited - for example, rewards/penalties can only be expressed based on the spatial locations of robots (or objects) at a particular time. However, the language is designed to be extensible, so this shortcoming can be addressed in future development (by the authors, or a third party).

 To aid the design process (particularly for non-expert users), future work may wish to consider the development of a graphical interface that can automatically generate SML files (e.g. via interactive placement of spatial entities), to avoid laborious textual specification directly in Xtext.

 Although the end-to-end design integration is validated in this article, there is no empirical evaluation of SML's usability, so it is unclear whether this new specification language actually makes the process of designing swarm behaviours significantly easier. The contribution would be significantly strengthened by conducting a user study like that presented in "An Experiment in Automatic Design of Robot Swarms: AutoMoDe-Vanilla, EvoStick, and Human Experts", to demonstrate the utility of SML in comparison to other methods like Buzz. ^1^

**Minor comments:**

 The figure captions are too brief and not sufficiently descriptive - it should be possible to understand what each figure represents in isolation, based on context from the caption.

 Figure 11 is excessively long - I suggest that you remove the XML comments, as they simply repeat the named tagged sections. Please also modify this example to use relative paths, instead of absolute paths specific to the author's personal filesystem.

 The code listings are quite difficult to read (especially the C++ code in Figure 12). Please consider using a condensed monospace font, reformatting the code, or including additional syntax highlighting to improve clarity. They should also be captioned as listings, rather than figures (like "Listing 1. Mission specification").

 Page 6 - "Phototaxis" should be italicised like the other items.

 Page 8 - Figure 3: "Perimetar" > "Perimeter"

 Page 10 - Under "Transition": "e.g.a" > "e.g., a"

 Page 11 - "s-boot" > "s-bot". Should this even say s-bot? The grammar presented in Figure 7 mentions only the e-puck and foot-bot (marXbot).

 Page 17 - "Fist" > "First"

 Page 17 - "xml" > "XML"

 Page 17 - "ARGOS" > "ARGoS"

Is the study design appropriate and does the work have academic merit?

Yes

Is the work clearly and accurately presented and does it cite the current literature?

Yes

If applicable, is the statistical analysis and its interpretation appropriate?

Not applicable

Are all the source data underlying the results available to ensure full reproducibility?

Yes

Are the conclusions drawn adequately supported by the results?

Yes

Are sufficient details of methods and analysis provided to allow replication by others?

Yes

Reviewer Expertise:

Swarm robotics

I confirm that I have read this submission and believe that I have an appropriate level of expertise to confirm that it is of an acceptable scientific standard.

## References

[1] : An Experiment in Automatic Design of Robot Swarms AutoMoDe-Vanilla, EvoStick, and Human Experts in ANTS 2014: Ninth International Conference on Swarm Intelligence. *Springer* .2014;25-37

# Response

**Published:** 2022-08-04

**Authors:** Darko Bozhinoski

**Affiliations:** 151927, The Netherlands

We are glad that the reviewer appreciated the work done. In the following, we address the comments point by point.

- We agree with the reviewer: the current implementation of SML is limited. It is indeed intended to be a proof of concept and opens the way to future developments. This is reflected also in the title of the article: “Towards an integrated automatic design process for robot swarms”.  As the reviewer points out, the language is extensible, and can be further developed, possibly by third parties. In this first, proof-of-concept implementation we decided to use spatial locations of robots for rewards/penalties due to the fact that in our analysis on a set of swarm missions, we noticed that most of the swarm missions measure success through an indicator that assesses the swarm being in a particular location at a specific time or by a mission objective that can be easily converted to this indicator. This definitely does not address all possible missions of interest, but covers a good share of them and, in our opinion, provides a clear idea of the potential of the proposal.
- Regarding the idea of developing a graphical interface to aid the design process and avoid laborious textual specification, we fully agree with the reviewer and we have plans to extend this contribution by introducing a graphical interface for swarm designers to design their missions as discussed in our conclusions. Yet, we would like to emphasise that the current textual specification of the swarm mission is already a significant step ahead with respect to what is described in the literature. Regarding the suggestion of conducting a user study to evaluate the SML usability, we would like to highlight that an evaluation of this kind is a huge endeavour and really rare in the literature. We feel that this deserves to be done as a separate study. We consider that an empirical evaluation of SML is an important future contribution that requires a definition of a protocol for evaluation based on objective qualitative and quantitative metrics that demonstrate the usefulness of our domain-specific language.
- We have reviewed and fixed all the other minor comments and typos highlighted by the reviewer.

We thank the reviewer for his valuable feedback.

# Peer review: Reviewer response for version 1

**DOI:** 10.21956/openreseurope.15113.r28418

**Published:** 2022-02-08

**Authors:** Adam Schroeder

**Affiliations:** 1 University of Toledo, Toledo, OH, USA

The main contribution of this work is the creation of a formal and standardized way to specify the requirements for a robot swarm mission and how the performance of a swarm can be quantitatively evaluated. Their tool is called swarm mission language (SML).  After the requirements for a mission have been specified in SML, existing software tools are used to simulate swarm performance, optimize performance, and eventually write a controller for a physical robot. Overall, SML in combination with existing software enables an automatic design process for robot swarms.

 The authors validated their tool and the use of an automatic design process with three example missions.

 This work fills a current research gap and creates a useful tool for a swarm designer. It is adopting automatic design principles that are used in other disciplines to the specific discipline of robot swarm design. The authors acknowledge which scenarios are currently supported by their tool and that it could be extended to support more.

 Communication within a swarm is one important design decision and it would be helpful for the authors to explicitly discuss this and how it is addressed with their tool. 

 In thinking of actual missions I would try to design a swarm for, it would be useful for the authors to discuss if and how their tool could handle (i) unknown or partially-known environments, where it would not be possible to specify their details in advance, (ii) dynamic objects in an environment, (iii) environments with non-discrete objects, e.g. a continuous distribution of food sources instead of discrete food sources, (iv) outdoor environments. Of course, even if these elements were supported by SML, they also would need to be supported in any simulator used.

Is the study design appropriate and does the work have academic merit?

Yes

Is the work clearly and accurately presented and does it cite the current literature?

Yes

If applicable, is the statistical analysis and its interpretation appropriate?

Not applicable

Are all the source data underlying the results available to ensure full reproducibility?

Yes

Are the conclusions drawn adequately supported by the results?

Yes

Are sufficient details of methods and analysis provided to allow replication by others?

Yes

Reviewer Expertise:

Swarm robotics

I confirm that I have read this submission and believe that I have an appropriate level of expertise to confirm that it is of an acceptable scientific standard.

# Response

**Published:** 2022-08-04

**Authors:** Darko Bozhinoski

**Affiliations:** 151927, The Netherlands

We are glad that the reviewer appreciated the  work we have done  so far. In the following, we will provide details and explanation about the concerns he raised.

 Regarding the question on how our work addresses communication  within a swarm, we would like to mention that AutoMoDe-Chocolate,  the automatic design method used in this work - does not work with  communication behaviours. In other works, we have developed  automatic design methods that design control software for robots  described by an extended reference model which include the ability to  communicate via light and infra-red signals. These design methods (AutoMoDe-Gianduja [1] and  AutoMoDe-TuttiFrutti [2]) are similar to AutoMoDe-Chocolate, but include modules that implement communication.The SML language can be extended to serve these other design methods.  Concerning the question on how SML can handle: (i) unknown or partially-known environments, (ii) dynamic objects in an environment, (iii) environments with non-discrete objects and (iv) outdoor environments, we would like to clarify that SML supports the specification of a class of missions where a probabilistic description of the environment is provided. The robot swarm performs the mission in this range of possible environments. In our current setup, SML enables specification of semi-structured partially-known environments, environments with non-discrete objects (region of food source) and the execution of the mission can happen in an indoor or outdoor environment. We thank the reviewer for his valuable feedback.

 [1] Hasselmann, Ken, and Mauro Birattari. "Modular automatic design of collective behaviors for robots endowed with local communication capabilities." PeerJ Computer Science 6 (2020): e291.

 [2] Garzón Ramos, David, and Mauro Birattari. "Automatic design of collective behaviors for robots that can display and perceive colors." Applied Sciences 10, no. 13 (2020): 4654.
